# An Advanced Lipid Metabolism System Revealed by Transcriptomic and Lipidomic Analyses Plays a Central Role in Peanut Cold Tolerance

**DOI:** 10.3389/fpls.2020.01110

**Published:** 2020-07-21

**Authors:** He Zhang, Chunji Jiang, Jingyao Ren, Jiale Dong, Xiaolong Shi, Xinhua Zhao, Xiaoguang Wang, Jing Wang, Chao Zhong, Shuli Zhao, Xibo Liu, Shibo Gao, Haiqiu Yu

**Affiliations:** Peanut Research Institute, College of Agronomy, Shenyang Agricultural University, Shenyang, China

**Keywords:** peanut, cold tolerance, transcript profiling, lipid metabolism, membrane lipid remodeling, fatty acid metabolism

## Abstract

Cold stress restricts peanut (*Arachis hypogaea* L.) growth, development, and yield. However, the specific mechanism of cold tolerance in peanut remains unknown. Here, the comparative physiological, transcriptomic, and lipidomic analyses of cold tolerant variety NH5 and cold sensitive variety FH18 at different time points of cold stress were conducted to fill this gap. Transcriptomic analysis revealed lipid metabolism including membrane lipid and fatty acid metabolism may be a significant contributor in peanut cold tolerance, and 59 cold-tolerant genes involved in lipid metabolism were identified. Lipidomic data corroborated the importance of membrane lipid remodeling and fatty acid unsaturation. It indicated that photosynthetic damage, resulted from the alteration in fluidity and integrity of photosynthetic membranes under cold stress, were mainly caused by markedly decreased monogalactosyldiacylglycerol (MGDG) levels and could be relieved by increased digalactosyldiacylglycerol (DGDG) and sulfoquinovosyldiacylglycerol (SQDG) levels. The upregulation of phosphatidate phosphatase (PAP1) and phosphatidate cytidylyltransferase (CDS1) inhibited the excessive accumulation of PA, thus may prevent the peroxidation of membrane lipids. In addition, fatty acid elongation and fatty acid β-oxidation were also worth further studied in peanut cold tolerance. Finally, we constructed a metabolic model for the regulatory mechanism of peanut cold tolerance, in which the advanced lipid metabolism system plays a central role. This study lays the foundation for deeply analyzing the molecular mechanism and realizing the genetic improvement of peanut cold tolerance.

## Introduction

Peanut (*Arachis hypogaea* L.), one of the most important grain legumes and a source of edible oils and proteins, is cultivated in tropical and subtropical regions of the world (Huang et al., 2019). In recent years, with increasing demand for peanuts, plantings have rapidly expanded in high-latitude areas such as northeast China (Bai et al., 2019). However, the heat condition in northeast China is poor and the extreme climate events such as low temperature caused by global climate change occur frequently, severely restricting peanut growth, development, productivity, and geographical distribution in temperate and high-elevation areas (Xiao and Song, 2011; Chen N. et al., 2014). Improper cold stress can increase the membrane permeability and membrane lipid peroxidation of peanut seedlings, resulting in electrolyte leakage (EL), excessive accumulation of reactive oxygen species (ROS), and substantial metabolic imbalance, especially involving respiration and photosynthesis (Vincent et al., 2018; Chang et al., 2019). In addition to morphological and physiological changes, cold stress can also lead to a series of complex signal transduction and transcriptional rearrangements (Zhang G. H. et al., 2019). These changes eventually incur damages at the whole-plant level, leading to the occurrence of chilling damage. Most temperate or hardy plants have evolved precise mechanisms to survive low temperature. Understanding the specific mechanism of peanut cold tolerance is the critical first step toward providing targets for genetic engineering of cold-tolerant peanut germplasm.

At the physiological level, malondialdehyde (MDA) and EL, the important signs of membrane damage, are negatively correlated with plant cold tolerance and have been used as indices for evaluating cold tolerance (Karami-Moalem et al., 2018). The current model of the signal transduction mechanism of cold tolerance in plants is that cold stress may first be sensed by plasma membrane proteins, such as calcium channels or associated proteins, resulting in calcium influx and leading to membrane fluidity changes and cytoskeleton rearrangement. Then, calcium-responsive protein kinases, including calcium-dependent protein kinases (CDPKs), calcineurin-B-like interacting protein kinases (CIPKs), and calcium-regulated receptor-like kinases (CRLKs), may mediate calcium signals to activate a mitogen-activated protein kinase (MAPK) cascade, which immediately regulates cold-regulated (COR) gene expression and plant cold stress tolerance (Kim et al., 2003; Almadanim et al., 2017; Yuan et al., 2018). COLD1, which regulates G-protein signaling by interacting with G-protein α subunit 1 (RGA1) and may regulate a calcium channel or be a cold-sensing calcium channel, is a transmembrane protein in the plasma membrane and endoplasmic reticulum (ER) and was recently reported to mediate cold stress sensing in rice (Ma et al., 2015). Ding et al. (2015) reported that cold stress can also activate sucrose nonfermenting 1-related protein kinase 2.6 (SnRK2.6)/open stomata 1 (OST1), which could interact with and phosphorylate transcription factors (TFs), such as calmodulin binding transcription activator (CAMTA) and ICE1/2, to activate the dehydration-responsive element-binding protein 1 (DREB1)/C-repeat-binding factors (CBF)-COR gene expression cascade.

However, despite much effort, except for the above conserved feature of cold tolerance in plants, little is known about other cold signal transduction pathways that are associated with gene expression changes. One of the latest discoveries in the field of cold signaling is the formation of the lipid second messenger, phosphatidic acid (PA), which shows to accumulate in suspension-cultured cells within minutes of cold stress (Cantrel et al., 2011; Peppino et al., 2017). In cold-induced signal transduction, PA responses have been mainly attributed to two pathways: the direct product of phospholipase D (PLD), which hydrolyses structural phospholipids such as phosphatidylcholine (PC) and phosphatidylethanolamine (PE), and the secondary product of phospholipase C (PLC) pathway, which first hydrolyzes polyphosphoinositides (PPIs) to diacylglycerol (DAG), and subsequently is phosphorylated to PA by diacylglycerol kinase (DGK) (Arisz et al., 2013; Tan et al., 2018). In addition, PA is the precursor to all phosphoglycerolipids as well as triacylglycerols (TAGs) and galactolipids, and its turnover is crucial in determining lipid metabolic fluxes and membrane compositions (Dubots et al., 2012). Lipid remodeling is another important contributor to cold tolerance, which has been proven in model plant *A. thaliana*, algae, and several crops, while there are specific changes among various plants (Gwak et al., 2014; Barrero-Sicilia et al., 2017; Noblet et al., 2017). In a non-plant system, in addition to PA, temperature stress also causes the generation of various lipid signals, including phosphatidylinositol (PI), sphingolipids, lysophospholipids, oxylipins, N-acylethanolamines, and many others (Suzuki et al., 2016). However, under cold stress, how these specific lipid species are generated and further affect cold tolerance in plants remains elusive.

Despite the evidence from model plants and crops that signal transduction, transcriptional reprogramming and lipid remodeling are essential to plant cold tolerance, there are only a handful of studies related to the cold responses of peanut, and the mechanism underlying peanut cold tolerance is poorly studied. Thus, in this study, we compared the variation in morphological and physiological parameters between tolerant and sensitive peanut varieties, and then identified the differences in gene expression profiles using RNA-seq. Finally, we determined the changes in free fatty acids (FFAs), membrane lipids and storage lipids using an LC-MS-based lipidomic platform and carried out integrated analyses with transcriptomic data. Based on information from multiple studies, we proposed a gene-metabolic network for the regulatory mechanism of peanut cold tolerance.

## Materials and Methods

### Plant Materials, Growth Conditions, and Treatments

Two peanut cultivars with contrasting responses to low temperature were used in this study: the cold-tolerant cultivar Nonghua5 (NH5) and the cold-sensitive cultivar Fuhua18 (FH18). These two cultivars were previously screened out of 68 landraces and cultivars from Northeast China according to their growth and morphological differences at the seedling stage under cold stress. NH5 had less damage than FH18 in response to low temperature, and NH5 can continue to survive normally after 7 d of cold treatment at 6°C, while the growth of FH18 was inhibited severely and the seedlings can hardly survive under cold stress. NH5 is the core parent used for breeding cold-tolerant peanut germplasm in Northeast China. FH18 is a representative peanut variety planted in large areas of northeast China. The seeds used in this study were obtained from the Peanut Research Institute, Shenyang Agricultural University, China.

Peanut seeds were surface sterilized with 3% sodium hypochlorite for 10 min, washed with distilled water five times, soaked in distilled water for 12 h, then placed in Petri dishes with moistened filter papers, and germinated in the dark at 28°C in a growth chamber. After 2 d, the germinated seeds were sown in round plastic pots filled with clean river sand and half strength Hoagland's solution and subsequently transferred to a climate chamber under a 16 h light (28°C)/8 h dark (23°C) cycle, a photosynthetic photon flux density of 700 µmol m^−2^ s^−1^ and a relative humidity of 70%, 10 seeds per plot.

Two-week-old seedlings were grouped into two groups. One group was transferred to another climate chamber maintained under 6°C, a 16 h/8 h cycle (light/dark), a photosynthetic photon flux density of 700 µmol m^−2^ s^−1^, and a relative humidity of 70%. The other group with the same developmental progression was used as a control and maintained under normal conditions. The second leaves from the treatment and control were collected at 0, 12, 24, 48, 72, 96, and 120 h, respectively, frozen in liquid nitrogen and stored at −80°C for measurements. All treatments were performed in three independent biological replicates.

### Morphological Parameters

After 7 d of cold stress, the seedlings under normal and cold conditions were sampled to measure plant height (PH), total leaf area (TLA), shoot (including leaves) fresh weight (SFW), root fresh weight (RFW), shoot dry weight (SDW), and root dry weight (RDW).

Fifteen seedlings for each treatment (5 seedlings per replicate) were selected, and the PH per plant was recorded. The TLA per plant was measured using an electronic area meter (Li-Cor3000, Li-Cor, Lincoln, NE, USA). Then, shoots and roots were separated, and the SFW and RFW were recorded. All plant tissues were dried at 105°C for 15 min and at 80°C for 72 h, and then the SDW and RDW were recorded.

### Determination of EL and MDA

The EL was determined using an electrical conductivity meter (DDSJ-308F, Shanghai, China) following Han et al. (2017). Small circular leaf samples were obtained by a 7-mm-diameter hole punch. Then, these circles were rinsed three times with deionized water and dried with filter papers. Twenty circles per replicate were put into a test tube with 20 ml deionized water. The initial electrical conductivity (EC1) of the washing solutions was measured after the samples were incubated at 25°C for 3 h. Then, the tubes were placed in a boiling water bath for 30 min, and the electrical conduction (EC2) was measured again after the solution cooled to room temperature. The EL was calculated using the formula EL = EC1/EC2×100.

The MDA content was determined according to Chen X. et al. (2014). Fresh leaves (0.5 g) were homogenized in 5 ml of 5% (m/v) trichloroacetic acid (TCA) and centrifuged at 12,000 g and 4°C for 10 min. The supernatant (2 ml) was mixed with an equal volume of 0.5% thiobarbituric acid (TBA). The mixture was placed in boiling water for 15 min and then instantly cooled in an ice bath and centrifuged at 10,000 g at 4°C for 10 min. The absorbance of the supernatant was measured at 450, 532, and 600 nm. The MDA content was calculated as follows, where Vt and Vs are the total volume of the extract solution and the volume of the extract solution contained in the reaction mixture, respectively, and m is the mass of the sample: MDA (nmol g^−1^ FW) = [6.45 × (A532-A600) − 0.56 × A450] × Vt/(Vs × m).

### Measurement of Photosynthetic Parameters

The net photosynthetic rate (Pn) was measured in the second leaves under cold and normal conditions using a portable photosynthetic system (LI-6400, Li-COR, Lincoln, NE, USA) at 70% relative humidity, a 370 µmol mol^−1^ CO_2_ concentration, and a 700 µmolm^−2^·s^−1^ PPFD (Sui et al., 2018).

The fluorescence parameter Fv/Fm and chlorophyll (Chl) fluorescence images of the second leaves under cold and normal conditions were analyzed after 30 min of dark adaptation using a Chl fluorescence imaging system FluorCam 7 (Photon Systems Instruments, Brno, Czech Republic) (Hantzis et al., 2018).

Chlorophyll (Chl) a, b, (a+b), and (a/b) contents were analyzed as previously described by Lichtenthaler and Wellburn (1983). Fresh leaves (0.1 g) were cut and placed in a 15 ml test tube with a 10 ml mixture of 95% acetone and absolute ethyl alcohol (1:1, v/v). The test tubes were then placed in dark conditions for 48 h until the leaves became white. The absorbance of the supernatant was measured at 663, 646, and 470 nm. The Chl contents were calculated as follows where V is the total volume of the extract solution:

$$\text{Chl}\;\text{a}\;(\text{mg g}{-}^{1}\;\text{FW})=(12.21\times \text{A}663-2.81\times \text{A}646)\times \text{V/FW}$$

$$\mathrm{Chl}\;\text{b}\;(\text{mg g}\;{-}^{1}\;\text{FW})=(20.13\times \text{A}646-5.03\times \text{A}663)\times \text{V/FW}$$

### Ultrastructural Observation

Small pieces (approximately 1 mm^2^) of fresh leaves after 0 (control) and 24 h of cold stress were fixed in 3% glutaraldehyde in 0.1 M phosphate buffer (pH 7.4) for 24 h at 4°C. Postfixation was conducted in 1% osmium tetroxide for 2 h, and the samples were then washed with phosphate buffer three times. Next, samples were dehydrated with an increasing series of ethanol (50% and 70%) and acetone (80% and 90%) for 15 min each. Then, the samples were dehydrated three times in 100% acetone for 30 min. The samples were soaked in a mix of propylene oxide and SPI-812 embedding medium. Finally, samples were immersed overnight in embedding medium. Ultrathin sections (50 nm) were cut using an ultramicrotome (Leica EM UC6 ultramicrotome, Japan) and collected on copper grids. Then, the sections were stained with uranyl acetate followed by lead citrate and examined using a transmission electron microscope (TEM, Joel JEM-1230, Japan).

### RNA Extraction, RNA-Seq, and Quantitative Real-Time RT-PCR

Total RNA was extracted from the second leaves of peanut seedlings subjected to 0 (control), 12, and 24 h of cold stress, respectively, using TRIzol reagent (Invitrogen). After quality inspection, a total of 20 μg of RNA from each sample was used for library construction. The process started with the synthesis of two cDNA strands, followed by the purification of the double-stranded cDNAs, and finally, the cDNA library was obtained by PCR enrichment. The library integrity was assessed using Qubit 2.0. The insert size was purified (AMPure XP system) and quantified using the Agilent high sensitivity DNA assay of the Agilent Bioanalyzer 2100 system. High-throughput sequencing was performed using the Illumina HiSeq system and was conducted in triplicate for each treatment. The raw data (raw reads) were filtered with the FASTQ_Quality_Filter tool from the FASTX-toolkit. Clean data were used for further analysis. After preprocessing the RNA-seq data, the reads were mapped to the peanut reference genome version Tifrunner.gnm1. ann1. CCJH. The sequence alignment generated by Tophat was then processed by Cufflinks software to assemble the alignments in the sequence alignment/map file into transcript fragments (transfrags).

Quantitative real-time RT-PCR (qRT-PCR) was performed to validate the RNA-seq results. Ten DEGs were randomly selected for qRT-PCR. All primers were designed using Primer 5.0 software (**Table S1**). The peanut actin gene (GenBank accession NC_037620) served as the internal control. The PCR was performed using SYBR Premix Ex Taq™ following the manufacturer's instructions (TaKaRa, Inc., Dalian, China) with a qRT-PCR amplification instrument (ABI 7500, USA). Each 10 μl reaction system contained 3.4 μl ddH2O, 5.0 μl SYBR^®^ Green Master Mix, 0.3 μl each primer, and 1.0 μl cDNA template. The PCR was initiated with a starting step of 95°C/60 s, followed by 40 cycles of 95°C/15 s and 55°C/30 s, and terminated at 72°C for 60 s. Three biological replicates were included in each treatment.

### Lipid Extraction

After 24 h of cold stress, the second peanut leaves from five plants in different pots were collected as one replication (the samples growing under normal conditions were used as controls), and the lipidomic analysis was performed with six replicates. Total lipid extraction was conducted according to previously reported methods (Narayanan et al., 2016) with slight modification. The cut peanut samples (approximately 200 mg) were quickly transferred into glass tubes with 3 ml preheated isopropanol [containing 0.01% butylated hydroxytoluene (BHT)] and held in a water bath for 15 min at 75°C. Then, 1.5 ml chloroform and 0.6 ultrapure water were added into the tubes, shaking at 150 g min^–1^ for 1 h. The extracted solution was then transferred into new glass tubes. The lipid extraction was repeated with 4 ml CHCl_3_/MeOH (2:1, v/v) containing 0.01% BHT at 150 g min^–1^ for 30 min and transferred again. The above steps were repeated until the samples became discolored. Then, the lipid extracts were combined, washed with 1 M KCl (1.0 ml), and centrifuged at 500 g for 5 min, and the water phase was discarded. Next, 2 ml ultrapure water was added to the extract and centrifuged at 500 g for 5 min, and the water phase was discarded. The solvent from the lipid extract was evaporated under a stream of N_2_ and stored at –80°C.

### Fatty Acid Composition Analysis

The prepared lipids were dissolved in 1 ml benzene/petroleum ether (1:1, v/v) and 1 ml methanol solution containing 0.4 M KOH), then 8 ml deionized water was added and shaken well. The supernatant was analyzed by a gas chromatograph system coupled with a mass spectrometer (GCMS-QP2010 Ultra, SHIMADZU, Japan) with a DB-Wax capillary column (Fan et al., 2017). A 1 μl aliquot of the analyte was injected. The GC conditions were as follows: split ratio of 1:20; injector and flame ionization detector temperature of 240°C; oven temperature programmed at 50°C for 2 min, increased 15°C min^−1^ to 150°C and then increased again at 6°C min^−1^ to 240°C, which was maintained for 4 min; carrier gas (H_2_) flow rate of 1 ml min^−1^. The mass spectrometry data were acquired in scan mode with the m/z range of 33–450 after a solvent delay of 3min.

### Lipidomic Analysis

Lipidomic analysis was performed on a 6460 triple quadruple electrospray ionization mass spectrometer (ESI/MS) coupled with a 1290 high-performance liquid chromatograph (Agilent, USA). The specific methods were previously described (Li et al., 2008; Gwak et al., 2014). The detailed method can be found in **Method S1**. For lipidomics analysis, six biological replicates were included. For each biological replicate, lipids were extracted in duplicate for independent LC/MS analysis.

### Statistical Analysis

All statistical analyses of physiological and lipidomic data were performed with SPSS 19.0 (SPSS Inc.) using one-way analysis of variance (ANOVA) and Tukey's test. Mean comparisons were performed using the least significant difference (LSD). *P<0.05 and **P<0.01 represent significant difference at the 0.05 and 0.01 level, respectively.

## Results

### Morphological Differences Between Cold Tolerant and Susceptible Varieties

In a previous study, 14-d-old peanut seedlings were exposed to 6°C for 7 d to examine the cold tolerance of 68 peanut cultivars by observing their phenotypes and measuring their morphological indexes. As shown in **Figure S1**, seedling leaves of FH18 were obviously dehydrated, wilted, and chlorotic, almost can't survive normally, while NH5 were only a little wilted in leaves and can continue to survive at 6°C low temperature. As for morphological characters, the plant height (PH), total leaf area (TLA), shoot fresh weight (SFW), root fresh weight (RFW), shoot dry weight (SDW), and root dry weight (RDW) of both NH5 and FH18 decreased. However, the magnitude of the decreases of FH18 were greater than those of NH5, especially for SFW, SDW, and RDW, which suggested that peanut cultivar FH18 was more sensitive to 6°C temperature exposure than NH5 and that the leaves and shoots of plants were more susceptible to cold stress than other tissues.

### Physiological and Ultrastructural Responses to Cold Stress

**Figure 1:**
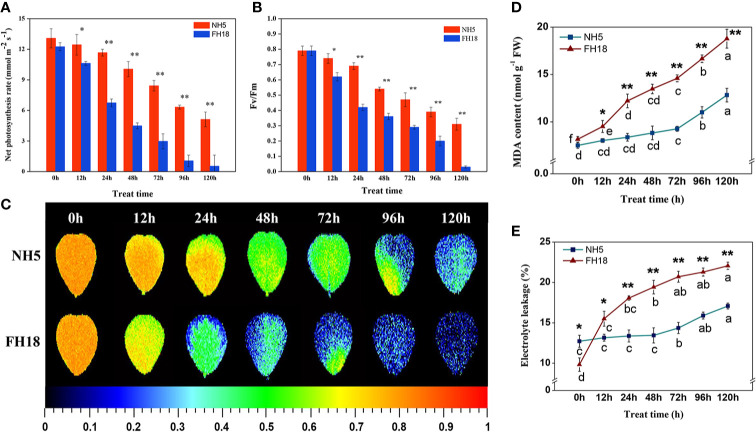
The physiological responses of NH5 and FH18 to continuous cold stress including **(A)** net photosynthetic rate (Pn), **(B, C)** maximum PSII quantum yield (Fv/Fm), **(D)** malondialdehyde (MDA) content, and **(E)** electrolyte leakage (EL). Error bars represent the SD of the means (three replicates). *Asterisk indicates significant difference as determined by the Student's t-test (*P < 0.05; **P < 0.01).

The photosynthetic capacity of NH5 and FH18 seedlings after 0, 12, 24, 48, 72, 96, and 120 h of cold stress were estimated by the net photosynthetic rate (Pn), Fv/Fm and Chl content, respectively. After continuous cold stress, the Pn and Fv/Fm were both decreased in the two peanut cultivars, while the levels in NH5 were higher than those in FH18. The differences between NH5 and FH18 reached an extremely significant level after 24 h of cold stress (**Figures 1A–C**). As shown in **Table S2**, Chl a, Chl b, Chl (a+b), and Chl (a/b) also declined continuously under cold stress, which may be the main factor behind leaf discoloration. The decreased Chl (a/b) suggested that Chl a may not be as stable as Chl b and be more easily decomposed and damaged under cold stress. According to the MDA contents and EL, membrane lipid oxidation and permeability also occurred in peanut under cold stress. After 120 h of cold stress, the MDA content in NH5 and FH18 increased by 109.54% and 169.97%, respectively. The EL in NH5 and FH18 increased by 38.41% and 135.65%, respectively. While, the changes of EL and MDA content in NH5 in the early stage of low temperature stress were not significant (**Figures 1D, E**).

**Figure 2:**
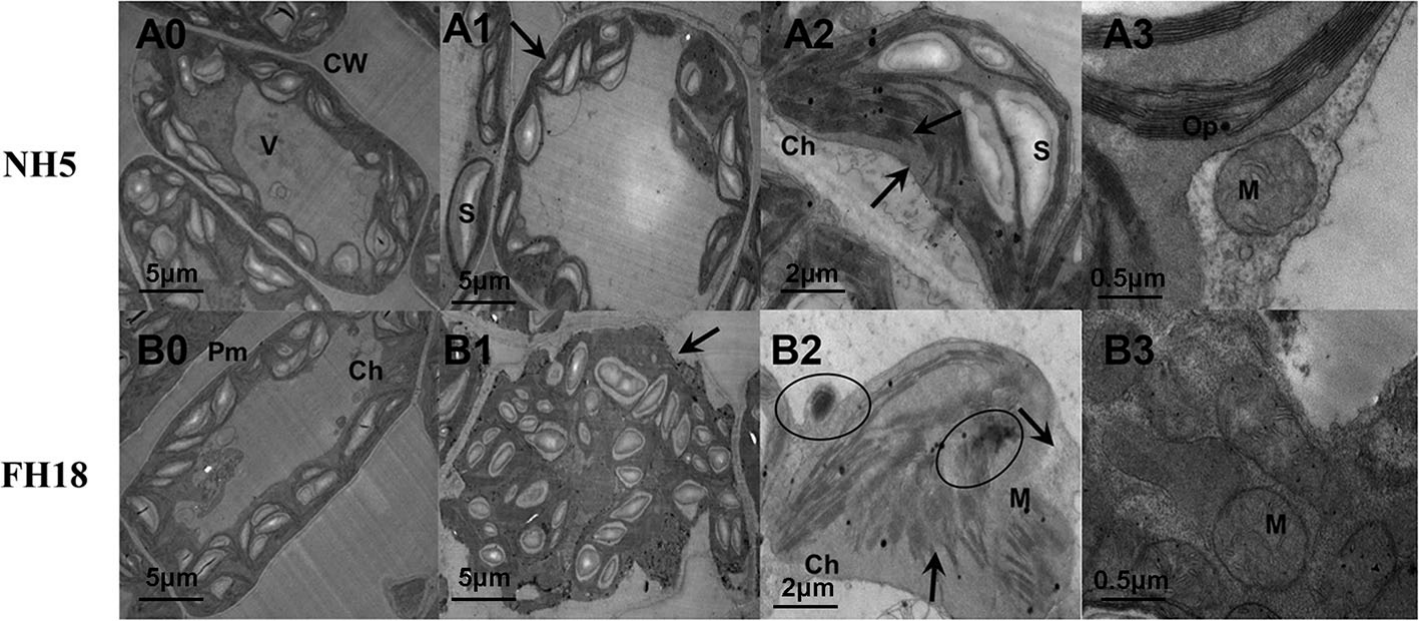
The ultrastructural changes of peanut leaves after 0 and 24 h cold treatments in NH5 (A0-A3) and FH18 (B0–B3). (A0, B0) The leaf cell under 0 h cold treatment. (A1, B1) The leaf cell under 24 h cold treatment. (A2, B2) The chloroplast under 24 h cold treatment. (A3, B3) The mitochondria under 24 h cold treatment. The arrows in (A1) and (B1) indicate the cell membrane. The arrows in (A2) and (B2) indicate the chloroplast membrane and granum. The circles in (B2) indicate the unknown floccular particles. Ch, chloroplast; CW, cell wall; M, mitochondria; Op, osmiophilic granules; Pm, plasma membrane; S, starch grain; V, vacuole. The black lines represent size marker bars.

These physiological responses indicated that NH5 was relatively stable, but FH18 was more severely damaged during the early stage (24 h) of cold stress. The reason for the decreased photosynthetic capacity, membrane lipid peroxidation and increased membrane permeability may due to damage to the membrane system and chloroplast structure under cold stress. Therefore, the subcellular structure of NH5 and FH18 leaves after 24 h of cold stress was observed. As shown in **Figure 2**, in NH5, the leaf cells, chloroplasts, and membrane system showed no significant changes. However, in FH18, the leaf cells were severely damaged, the cell walls were contorted, and chloroplasts became swollen and twisted, filling the entire cell chamber. Moreover, massive amounts of unknown floccular particles accumulated on the membrane, and severe damage occurred to the plasma membrane and chloroplast membrane structures with low levels of thylakoid stacking and without the typical grana.

### Genome-Wide Gene Expression Profiling in Peanut Under Cold Stress

To systematically reveal the specific cold stress responses at the genomic level and the underlying molecular regulatory mechanism, the transcriptional profiling of NH5 and FH18 exposed to 0, 12, and 24 h of cold stress were compared using Illumina RNA-seq (**Table S3**). The RNA-Seq data has been submitted to the online SRA (Sequence Reads Archive) database with the accession number PRJNA602777. A total of 36,944 effectively expressed genes [fragments per kilobase of transcript per million fragments mapped (FPKM) ≥1] were obtained (**Table S4**). Then, based on the statistical test [fold change (FC)≥2 and false discovery rate (FDR)<0.01], the differentially expressed genes (DEGs) in NH5 and FH18 were identified (**Table S5**). Of these, 3910 (2428 showing upregulation and 1482 showing downregulation) and 3702 (2070 upregulation and 1632 downregulation) had ≥ 2-FC in transcription activity relative to that at 28°C at any point, that is, continuously differential expression (CDEGs) during the early stage of cold stress in NH5 and FH18, respectively (**Figure S2**; **Tables S6** and **S7**).

**Figure 3:**
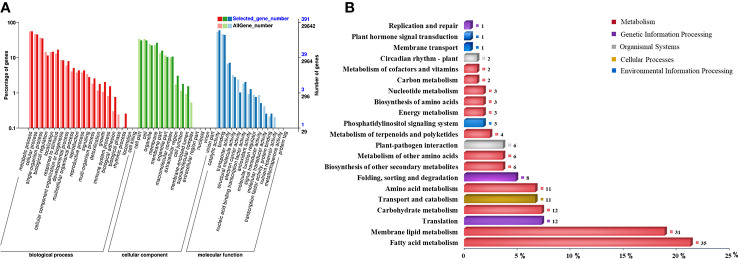
Functional enrichment analysis of 759 continuously differentially expressed genes (CDEGs) in “putative cold tolerance gene set” during the early stage of cold stress. **(A)** Enriched gene ontology (GO) classification. **(B)** Kyoto Encyclopedia of Genes and Genomes (KEGG) classification.

Among all the CDEGs, 569 (277 upregulation and 292 downregulation) were expressed only in NH5 (**Figure S2**; **Table S8**), and 2358 (1534 upregulation and 824 downregulation) were commonly expressed in both cultivars (**Figure S2**; **Table S9**). Moreover, based on the FC values, the differential expression level of 190 (158 upregulation and 32 downregulation) of 2358 CDEGs was significantly higher in NH5 than in FH18 at any point [FC(NH5/FH18) ≥2] (**Table S10**), which comprised the “putative cold tolerance gene set” together with the 569 CDEGs and may play a central role in peanut cold tolerance. These 759 CDEGs were enriched in 39 known GO terms (**Table S11**), and more were classified into the “biological process” category than the “cellular component” or “molecular function” categories (**Figure 3A**). The “metabolic process” in the biological process category was most significantly enriched. The top enriched GO terms in the cellular component category included “cell”, “organelle,” and “membrane”. The top enriched GO terms in the molecular function category included “catalytic activity” and “binding”. Furthermore, the biological metabolic process was analyzed in detail by the Kyoto Encyclopedia of Genes and Genomes (KEGG). A total of 147 CDEGs were enriched in 72 KEGG pathways (**Table S12**), of which “fatty acid metabolism”, “membrane lipid metabolism”, “translation”, “carbohydrate metabolism,” and “transport and catabolism” had the highest number of CDEGs (**Figure 3B**).

### Identification of Key DEGs in Lipid Metabolic Pathways for Cold Tolerance

**Figure 4:**
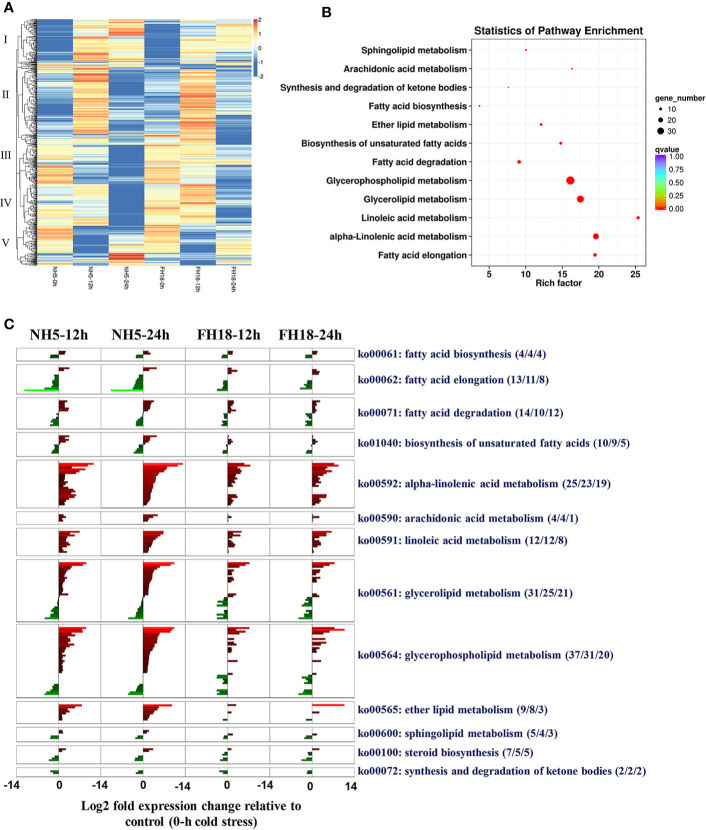
Identification and analyses of CDEGs involved in lipid metabolic pathways during the early stage of cold stress in NH5 and FH18. **(A)** The heat map of expression profiles of 651 genes involved in lipid metabolic pathways under 0, 12, and 24 h cold stress based on the log2–median transformed FPKM averages of three independent biological replications, the expression pattern was categorized into five groups. Red indicates high expression, white indicates intermediate expression, and blue indicates low expression. **(B)** The statistics of KEGG pathway enrichment of 111 CDEGs related to lipid metabolism during the early stage of cold stress. **(C)** The transcriptional changes of 111 CDEGs involved in 13 lipid metabolic pathways during the early stage of cold stress in NH5 and FH18. Aside pathway IDs and descriptions are indicated under the format (X/Y/Z), X indicates the number of CDEGs in the KEGG pathway, Y indicates the number of CDEGs significantly differentially expressed only in NH5 and Z indicates the number of CDEGs differentially expressed only in FH18.

Transcriptional profiling of two peanut cultivars indicated that lipid metabolism may play a central role in peanut cold tolerance; therefore, we further focused our analyses on the changes in transcription activity of a compiled list of 651 genes involved in lipid metabolism (**Table S13**), which were identified based on a homology search to known lipid metabolism genes in the higher model plant *Arabidopsis thaliana* reported in Aralip website (http://aralip.plantbiology.msu.edu/pathways/pathways). According to the clustering heat map, 111 CDEGs (74 showing upregulation and 37 showing downregulation) contained in clusters I and III were identified (**Figure 4A**; **Table S14**) and categorized into 13 lipid metabolic pathways (**Figure 4B**). The transcriptional profiles of 111 CDEGs in FH18 were significantly different from those in NH5, mainly manifested as smaller quantities and lower expression levels (**Figure 4C**). In NH5, CDEGs involved in “alpha-linolenic acid metabolism”, “linoleic acid metabolism”, “glycerolipid metabolism”, and “glycerophospholipid metabolism” were abundant, and most of them were highly upregulated, while CDEGs involved in “fatty acid elongation” were downregulated.

Of all the 111 CDEGs, 37 were expressed only in NH5, and 22 of 56 CDEGs showed significantly higher expression in NH5 than in FH18 (**Table S14**). We further characterized the functional annotation of these 59 lipid-related CDEGs involved in peanut cold tolerance (**Table S15**). Under cold stress, genes involved in the *de novo* synthesis of the storage lipid TAG were all significantly upregulated, including two glycerol-3-phosphate acyltransferase (*GPAT2*) genes, one lysophosphatidyl acyltransferase (*LPAAT*) gene, two phosphatidate phosphatase (*PAP1*) genes, and one diacylglycerol acyltransferase (*DGAT1*) gene, which may directly lead to the accumulation of DAG and TAG. DAG is a lipid intermediate that is the substrate for membrane lipid synthesis. In membrane-lipid metabolism pathways, genes responsible for the generation of PA, such as *PLDs* (*PLDζ*, *PLDζ1*, and *PLDζ2*) and DGK5, were also upregulated. PA can form CDP-DAG by the catalysis of phosphatidate cytidylyltransferase (*CDS1*) and then produce a small quantity of PI and cardiolipin (CL) by CDP-diacylglycerol–inositol 3-phosphatidyltransferase (*PIS1*) and cardiolipin synthase (*CRLS*), respectively. Here, five genes encoding monogalactosyl diacylglycerol synthase (*MGD*), digalactosyl diacylglycerol synthase (*DGD*), and sulfoquinovosyl diacylglycerol (SQDG) synthase (*SQD*) in galactolipid synthesis pathways were all upregulated. MGD and DGD1 catalyzed the formation of digalactosyldiacylglycerol (DGDG) from monogalactosyldiacylglycerol (MGDG) and *SQD2* catalyzed the formation of SQDG.

Most of the other CDEGs were involved in fatty acid metabolism pathways. Under cold stress, two key genes encoding acyl-CoA oxidase (*ACOX1*) and acetyl-CoA acyltransferase (*ACAA1*) were upregulated and promoted the β-oxidation of fatty acids. Then, acyl CoA with carbon chains of various lengths was hydrolyzed to FFAs by acyl-CoA thioesterase (*ACOT*). The β-oxidation of fatty acids is also the central process in the α-linolenic acid metabolic pathway. With the activation of β-oxidation and the upregulation of other key genes, including six lipoxygenase (*LOX*) genes, four allene oxide synthase (*AOS*) genes, one allene oxide cyclase (*AOC*) gene, and four jasmonate O-methyltransferase (*JMT*) genes, the α-linolenic acid metabolism and jasmonic acid (JA) biosynthesis were significantly activated. In contrast, the genes mapped to the fatty acid elongation pathway, such as 3-ketoacyl-CoA synthase (*KCS*), very-long-chain 3-oxoacyl-CoA reductase (*KCR*), very-long-chain (3R)-3-hydroxyacyl-CoA dehydratase (*HACD*), and very-long-chain enoyl-CoA reductase (*TER*) were all downregulated to inhibit the production of very long-chain fatty acids.

**Figure 5:**
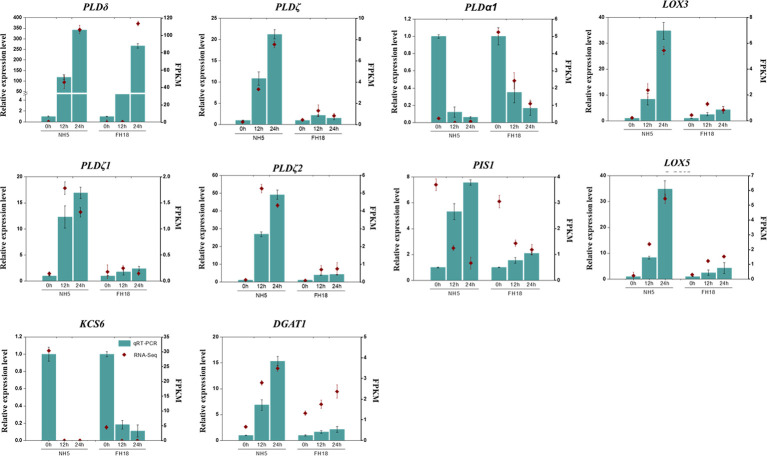
Quantitative real-time PCR (qRT-PCR) validation and RNA-seq data of ten selected DEGs in NH5 and FH18.

To verify the transcriptomic profile based on gene expression levels, ten CDEGs (*KCS6*, *DGAT1*, *LOX3*, *LOX5*, *PIS1*, *PLDα1*, *PLDδ*, *PLDζ*, *PLDζ1, and PLDζ2*) with differential expression patterns were randomly selected from the putative cold tolerant gene set for quantitative RT-PCR analysis (**Figure 5**). As expected, among all the selected genes, the relative expression levels of *DGAT1*, *LOX3*, *LOX5*, and *PLDζ/ζ1/ζ2* significantly increased in NH5 but were relatively stable in FH18. *PLDδ* was markedly upregulated and KCS6 was downregulated both in NH5 and FH18, which showed the same expression patterns with RNA-Seq analysis.

### Quantitative Changes in Lipid Composition Induced by Cold Stress

**Figure 6:**
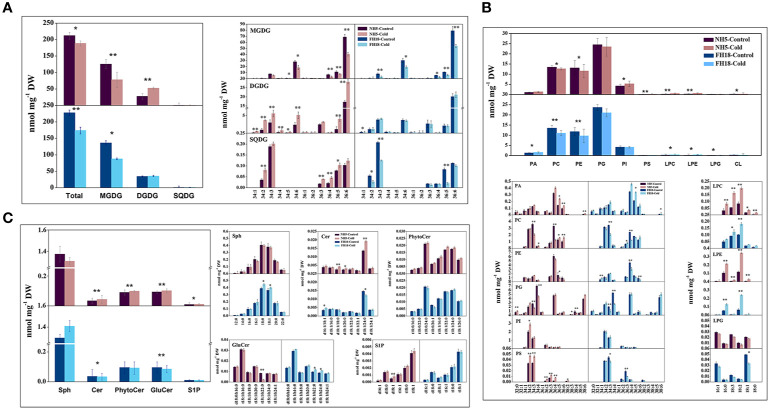
The changes of galactolipid, phospholipid and sphingolipid composition after 0 and 24 h cold treatments in NH5 and FH18. **(A)** The changes of total lipid and galactolipid composition. **(B)** The changes of phospholipid composition. **(C)** The changes of sphingolipid composition. Values are the means ± SD of six independent biological samples. *Asterisk indicates significant difference as determined by the Student's t-test (*P < 0.05; **P < 0.01). Cer, ceramide; CL, cardiolipin; DGDG, digalactosyldiacylglycerol; GluCer, glucosylceramide; LPC, lyso-phosphatidylcholine; LPE, lyso-phosphatidylethanolamine; LPG, lyso-phosphatidylglycerol; MGDG, monogalactosyldiacylglycerol; PA, phosphatidic acid; PC, phosphatidylcholine; PE, phosphatidylethanolamine; PG, phosphatidylglycerol; PhytoCer, phytoceramide; PI, phosphatidylinositol; PS, phosphatidylserine; Sph, sphingasine; SQDG, sulfoquinovosyl diacylglycerol; S1P, sphingosine-1-phosphate.

**Figure 7:**
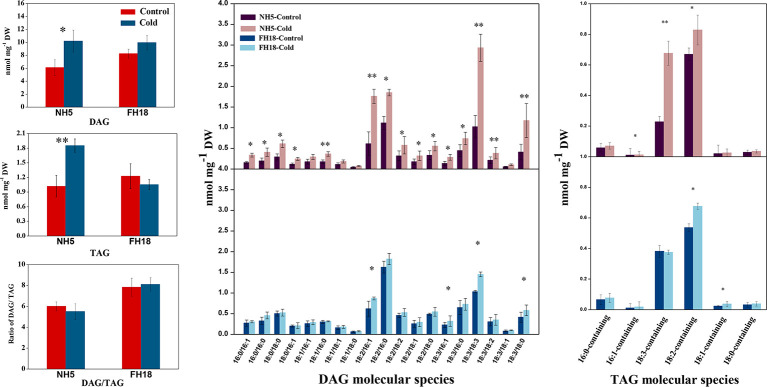
The changes of lipid intermediate and storage lipid composition after 0 and 24 h cold treatments in NH5 and FH18. Values are the means ± SD of six independent biological samples. *Asterisk indicates significant difference as determined by the Student's t-test (*P < 0.05; **P < 0.01). DAG, diacylglycerol; TAG, triacylglycerol.

To investigate whether the differential expression of lipid metabolism genes was reflected in differential lipid abundances, the lipidomes of NH5 and FH18 were compared quantitatively during the early stage of cold stress. Specifically, phospholipids, galactolipids, sphingolipids, diacylglycerols, and TAGs were targeted. In our study, a total of 20 headgroup classes of lipids and 168 molecular lipid species were detected (**Figures 6** and **7**; **Table S16**). The total lipid contents of NH5 and FH18 under control conditions were 219.27 and 237.16 nmol mg^−1^ DW but decreased by 7.32% and 22.14% after cold stress, respectively (**Figure 6A**).

Galactolipids, including MGDG, DGDG, and SQDG, are the main constituents of the chloroplast membranes and photosynthetic complexes, accounting for approximately 70% of the total lipids. The profiling of galactolipids provided consistent data with the transcriptomic analysis that indicated a decrease in the integrity of chloroplast membrane. Indeed, the MGDG contents of both cultivars were significantly decreased, which was mainly caused by the significant reduction in C34:6- (total number of acyl carbon atoms: number of double bounds) and C36:6-MGDG. However, in NH5, almost all molecular species of DGDG and SQDG were significantly increased, which significantly inhibited the reduction of chloroplast membrane lipid and maintained the integrity of the chloroplast membrane to a certain extent, thereby inhibiting the significant reduction in photosynthesis. Besides, the transfer of acyl chains from C34:6-, C36:5-, and C36:6-MGDG to C34:6-, C36:5-, and C36:6-DGDG was the main reason for decreased MGDG but increased DGDG in NH5 (**Figure 6A**).

Phospholipids, a large family of glycerolipids, accounted for approximately 23–27% of the total lipids. Of which phosphatidyl glycerol (PG), PC, and PE were the most abundant, contributing to approximately 11%, 6%, and 5.5% of the overall lipidome, respectively, but their contents were all reduced, especially in FH18, with the C34:4 and C36:4 species contributing the most to the decline in PC and PE. Conversely, the levels of lyso-phospholipids including lyso-PC (LPC), lyso-phosphatidylethanolamine (LPE), and lyso-phosphatidylglycerol (LPG) significantly increased in both two varieties, suggesting that the increased activities of PLDs (**Figure 5**) promoted the lipid-hydrolytic pathway under cold stress. Other phospholipids, including PA, PI, phosphatidylserine (PS), and CL, were present in minor amounts ranging from 0.03 to 2.6% of the total lipids, and their contents were also increased in NH5. Notably, a significantly higher level of PA in FH18 was observed (**Figure 6B**). Compared with NH5, the PA content in FH18 increased by 1.21 times under cold stress. The major contributors to increased PA were C36:5 and C36:6 in NH5 but C36:4 and C36:5 in FH18.

Sphingolipids were also the main structural lipids, which were composed of five classes in peanut: sphingomyelin (Sph), ceramide (Cer), phytoceramide (PhytoCer), glucosylceramide (GluCer), and sphingosine-1-phosphate (S1P). In response to cold stress, Cer, PhytoCer, and GluCer increased, and Sph and S1P decreased in NH5, while the trends of these five SPL classes in FH18 completely contrasted with those in NH5 (**Figure 6C**). Only a few molecular species changed by cold stress in both two cultivars. d18:1/24:0-Cer was the major component that determined the increase or decrease of Cer. And t18:1/h22:0-GluCer was most affected by cold stress in GluCer in NH5.

In addition to membrane lipids, small amounts of the intermediate lipid DAG and the storage lipid TAG were also detected. There were 19 DAG molecular species in peanut leaves. The contents of DAGs with acyl chains of 18:2/16:1, 18:2/16:0, 18:3/18:3, and 18:3/18:0 were drastically higher in NH5, which were the major contributors to the increased DAG (**Figure 7**). TAG was composed of a few molecular species in peanut leaves, of which 18:2-containing and 18:3-containing TAGs were the main component. The TAG content was increased in NH5 but decreased in FH18, and a decreased DAG-TAG ratio was observed in the cold-tolerant cultivar NH5.

### Changes in Membrane Lipid Unsaturation Induced by Cold Stress

The dramatic changes in lipid molecular species may influence the fatty acid classes connected to glycerin molecules, resulting in an alteration of membrane lipid unsaturation. As shown in **Table 1**, the unsaturated double bond index (DBI) of the total membrane lipids increased by 7.92% and decreased by 13.25% in NH5 and FH18, respectively, indicating that NH5 may adapt to cold stress by increasing the membrane lipid unsaturation level. The lower DBI of MGDG was the main reason for the decreased membrane lipid unsaturation. Among all membrane lipid classes, DGDG, PA, PS, SQDG, and PI showed larger relative changes (RC) in DBI ranging from 109.41% to 42.03% and were the major contributors to the increase in total membrane lipid unsaturation, The DBI of sphingolipid classes Sph and GluCer also increased, while sphingolipid was only a minor constituent and contributed little to the DBI of the total membrane lipids.

**Table 1:** Double-bond index (DBI) of membrane lipids after 0 and 24 h cold treatments in NH5 and FH18.

Lipid class	Double bond index (DBI)				Relative change (RC)	
	NH5-Control	NH5-Cold	FH18-Control	FH18-Cold	NH5	FH18
MGDG	3.3534 ± 0.07^a^	2.8792 ± 0.13^a^	3.2965 ± 0.42^a^	2.2921 ± 0.64^a^	−0.1414	−0.3047
DGDG	0.6750 ± 0.04^b^	1.4135 ± 0.09^a^	0.7822 ± 0.08^a^	1.0660 ± 1.03^a^	1.0941	0.3628
SQDG	0.0084 ± 0.00^b^	0.0125 ± 0.02^a^	0.0086 ± 0.04^a^	0.0072 ± 0.01^a^	0.4881	−0.1628
PA	0.0195 ± 0.01^b^	0.0339 ± 0.03^a^	0.0174 ± 0.07^b^	0.0262 ± 0.02^a^	0.7385	0.5057
PC	0.2061 ± 0.04^a^	0.2271 ± 0.05^a^	0.2183 ± 0.04^a^	0.2349 ± 0.07^a^	0.1019	0.0760
PE	0.1911 ± 0.05^a^	0.1921 ± 0.01^a^	0.2279 ± 0.02^a^	0.2179 ± 0.05^a^	0.0052	−0.0439
PG	0.3981 ± 0.03^a^	0.4720 ± 0.02^a^	0.4464 ± 0.03^a^	0.4824 ± 0.06^a^	0.1856	0.0806
PI	0.0069 ± 0.01^b^	0.0098 ± 0.04^a^	0.0064 ± 0.02^a^	0.0086 ± 0.02^a^	0.4203	0.3438
PS	0.0010 ± 0.00^b^	0.0017 ± 0.00^a^	0.0013 ± 0.01^a^	0.0014 ± 0.01^a^	0.7000	0.0769
CL	0.0109 ± 0.00^a^	0.0130 ± 0.02^a^	0.0083 ± 0.03^a^	0.0117 ± 0.03^a^	0.1927	0.4096
Sph	0.0024 ± 0.00^a^	0.0035 ± 0.01^a^	0.0028 ± 0.01^a^	0.0030 ± 0.01^a^	0.4583	0.0714
Cer	0.0002 ± 0.00^a^	0.0003 ± 0.00^a^	0.0002 ± 0.00^a^	0.0003 ± 0.00^a^	0.2827	0.3074
PhytoCer	0.0003 ± 0.00^a^	0.0004 ± 0.00^a^	0.0003 ± 0.00^a^	0.0004 ± 0.01^a^	0.3333	0.3333
GluCer	0.0004 ± 0.00^a^	0.0006 ± 0.00^a^	0.0004 ± 0.00^a^	0.0004 ± 0.00^a^	0.5000	0.0000
S1P	0.0000 ± 0.00^a^	0.0000 ± 0.00^a^	0.0000 ± 0.00^a^	0.0000 ± 0.00^a^	0.0000	0.0000
Total lipids	4.8736 ± 0.10^a^	5.2594 ± 0.54^a^	5.0170 ± 0.31^a^	4.3524 ± 0.18^a^	0.0792	−0.1325

DBI = (Ʃ [N × mol % lipid])/100, N is the total number of double bonds in the two fatty acid chains of each membrane lipid molecule. The DBI is the average number of double bonds in the fatty acid chains of lipid molecular species, a high DBI indicates the presence of more highly unsaturated membrane lipids, and vice versa. Data are the means ± SD of six independent biological samples. Means denoted by the same letter do not differ significantly according to Tukey's test (p < 0.05).

### Changes in Free Fatty Acid Components Induced by Cold Stress

A total of 22 FFA components were detected in peanut leaves (**Table 2**). Although the main FFA classes were similar, there was great diversity in their contents between the two cultivars under cold stress. The total fatty acid (TFA) contents in NH5 and FH18 were increased by 4% and 0.7%, respectively. At the same time, the content of saturated (SFAs) and monounsaturated fatty acids (MUFAs), including C16:0, C16:1, C18:0, and C18:1, obviously decreased in NH5 but were almost unchanged in FH18. The polyunsaturated fatty acids (PUFAs) C18:2 and C18:3 markedly increased in NH5 but increased more slowly in FH18. In general, NH5 accumulated more PUFAs (increased by 23%) than FH18 (increased by 2%) and exhibited the genetic characteristic of having more PUFAs during the long-term adaptation to low temperature. The main reason for this finding may be that NH5 can increase the ratio of PUFAs to SFAs (increased by 36%) by the rapid synthesis of PUFAs (C18:2 and C18:3).

**Table 2:** The changes of fatty acid components after 0 and 24 h cold treatments in NH5 and FH18.

Fatty acid (ng mg ^−1^)	NH5		FH18		Relative change	
	Control	Cold	Control	Cold	NH5	FH18
C14:0	0.39 ± 0.01^a^	0.37 ± 0.05^a^	0.20 ± 0.01^b^	0.37 ± 0.06^a^	−0.10	0.87
C14:1	0.95 ± 0.06^a^	0.91 ± 0.10^a^	0.37 ± 0.04^a^	0.34 ± 0.04^a^	−0.08	−0.08
C16:0	151.87 ± 9.69^a^	131.96 ± 11.21^b^	149.52 ± 10.68^a^	144.61 ± 12.59^a^	−0.17	−0.04
C16:1	51.29 ± 3.21^a^	41.41 ± 5.12^b^	51.14 ± 2.97^a^	53.16 ± 6.71^a^	−0.22	0.03
C18:0	213.37 ± 7.54^a^	195.57 ± 15.25^a^	194.53 ± 9.23^a^	195.45 ± 10.04^a^	−0.12	0.00
C18:1	59.60 ± 2.48^a^	51.95 ± 4.63^b^	58.82 ± 5.59^a^	58.31 ± 4.97^a^	−0.16	−0.02
C18:2	83.50 ± 4.44^b^	117.37 ± 9.13^a^	79.13 ± 4.67^b^	99.19 ± 6.42^a^	0.35	0.24
C18:3	164.18 ± 5.86^b^	211.32 ± 7.99^a^	166.05 ± 11.35^a^	186.73 ± 9.68^a^	0.24	0.12
C20:0	2.73 ± 0.54^a^	1.89 ± 0.22^b^	2.07 ± 0.12^a^	1.39 ± 0.20^b^	−0.34	−0.33
C20:1	0.53 ± 0.09^b^	1.14 ± 0.13^a^	0.67 ± 0.06^a^	0.55 ± 0.06^ab^	1.05	−0.18
C20:2	0.15 ± 0.03^b^	0.27 ± 0.08^a^	0.22 ± 0.03^a^	0.20 ± 0.01^a^	0.75	−0.09
C20:3	56.72 ± 2.96^a^	62.60 ± 5.54^a^	1.08 ± 0.14^a^	0.80 ± 0.02^b^	0.06	−0.27
C20:4	81.03 ± 4.17^a^	84.53 ± 3.69^a^	65.95 ± 7.26^a^	33.33 ± 3.68^b^	0.00	−0.50
C20:5	0.31 ± 0.11^b^	0.38 ± 0.02^a^	0.41 ± 0.03^a^	0.35 ± 0.03^ab^	0.21	−0.17
C22:0	1.78 ± 0.25^a^	2.11 ± 0.17^a^	0.21 ± 0.01^a^	0.20 ± 0.01^a^	0.14	−0.05
C22:1	0.34 ± 0.04^b^	0.55 ± 0.10^a^	0.43 ± 0.11^a^	0.42 ± 0.04^a^	0.55	−0.03
C22:2	0.41 ± 0.06^a^	0.48 ± 0.06^a^	0.49 ± 0.06^a^	0.43 ± 0.10^a^	0.10	−0.13
C22:4	1.17 ± 0.10^a^	1.34 ± 0.21^a^	1.36 ± 0.27^a^	1.21 ± 0.31^a^	0.10	−0.12
C22:5	2.53 ± 0.15^b^	3.04 ± 0.26^a^	2.97 ± 0.33^a^	2.64 ± 0.19^a^	0.15	−0.12
C22:6	2.55 ± 0.08^a^	2.89 ± 0.14^a^	2.99 ± 0.16^a^	2.63 ± 0.12^a^	0.09	−0.13
C24:0	1.89 ± 0.24^a^	1.46 ± 0.33^b^	1.47 ± 0.08^b^	3.48 ± 0.20^a^	−0.26	1.35
C24:1	0.63 ± 0.04^a^	0.72 ± 0.10^a^	0.67 ± 0.05^a^	0.60 ± 0.05^a^	0.10	−0.12
Total	877.90 ± 13.69^a^	914.26 ± 15.25^a^	780.77 ± 12.53^a^	786.40 ± 14.45^a^	0.04	0.007
PUFA	392.55 ± 13.17^b^	484.22 ± 6.74^a^	320.65 ± 11.33^a^	327.51 ± 7.42^a^	0.23	0.02
SFA	372.02 ± 10.12^a^	333.35 ± 3.97^a^	348.00 ± 9.71^a^	345.51 ± 4.95^a^	−0.10	−0.01
PUFA/SFA	1.06 ± 0.12^b^	1.45 ± 0.14^a^	0.92 ± 0.06^a^	0.95 ± 0.05^a^	0.36	0.03

The 2nd leaf from each peanut seedling after 0 and 24 h cold treatments was harvested for the determination of fatty acid content (ng mg^−1^). Data are the means ± SD of six independent biological samples. Means denoted by the same letter do not differ significantly according to Tukey's test (p < 0.05). PUFA, polyunsaturated fatty acid; SFA, saturated fatty acid.

### The Gene-Metabolite Network Revealed the Molecular Regulation Mechanism of Lipids Metabolism in Peanut Cold Tolerance

**Figure 8:**
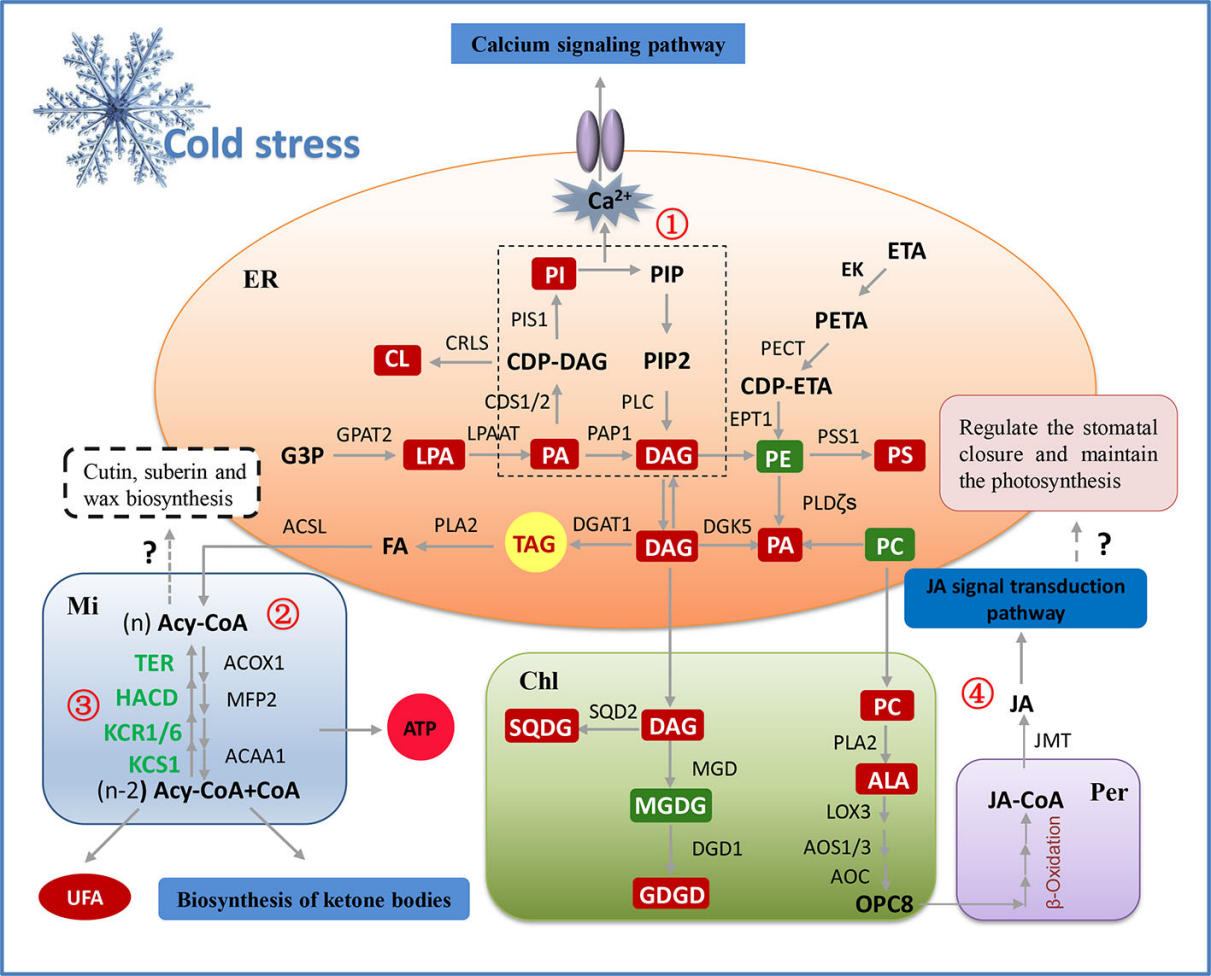
The model illustrating potential regulation mechanism of membrane lipid metabolism in peanut cold tolerance. The glycerolipid, glycerophospholipid and fatty acid metabolic pathways were the significant pathways altering in response to peanut cold tolerance, which were depicted as fully as possible based on the integration of lipidomic and transcriptomic responses. The red boxes represent the increased metabolites and the green boxes represent the decreased metabolites. Except the genes (green words) involved in fatty acid elongation (③), all the genes in this figure were up-regulated. Besides to alter membrane lipid composition and membrane lipid remodeling, lipid classes can also adapt to cold stress through other metabolic processes. For example, accumulated PI can activate the phosphatidylinositol signaling pathway, further increased the concentration of Ca^2+^ and activated the Ca^2+^ channel to regulate the expression of downstream cold-tolerant genes (①). The storage lipid TAG can be hydrolyzed by PLA_2_ to produce the FFA and leading to the activation of fatty acid degradation and fatty acid elongation pathways. The fatty acid degradation may provide enough energy for peanut to adapt to cold stress, as well as produce more polyunsaturated short-chain fatty acids to maintain the membrane fluidity (②). The fatty acid elongation may be related to the biosynthesis of plant wax, while the explicit mechanism needs to be further studied (③). Also, the structural lipids may be degraded under cold stress and synthesize the JA through α-linolenic acid metabolism and β-oxidation of fatty acid, then to activate the JA signal transduction that may regulate the stomatal closure and maintain the photosynthesis, but this hypothesis needs to be further validated in peanut (④). ACAA1, acetyl-CoA acyltransferase; ACOX1, acyl-CoA oxidase; ACSL, long-chain acyl-CoA synthetase; Acy-CoA, acyl-coenzyme A; ALA, α-linolenic acid; AOC, allene oxide cyclase; AOS, allene oxide synthase; CDS1/2, phosphatidate cytidylyltransferase; Chl, chloroplast; CL, cardiolipin; CRLS, cardiolipin synthase; DAG, diacylglycerol; DGAT1, diacylglycerol acyltransferase; DGD1, digalactosyl diacylglycerol synthase; DGDG, digalactosyldiacylglycerol; DGK5, diacylglycerol kinase; EK, ethanolamine kinase; EPT1, ethanolamine phosphotransferase; ER, endoplasmic reticulum; ETA, ethanolamine; FA, fatty acid; G3P, glycerol-3-phosphate; GPAT2, glycerol-3-phosphate acyltransferase; HACD, very-long-chain (3R)-3-hydroxyacyl-CoA dehydratase; JA, jasmonic acid; JMT, jasmonate O-methyltransferase; KCR1/6, very-long-chain 3-oxoacyl-CoA reductase; KCS1, 3-ketoacyl-CoA synthase; LOX3, lipoxygenase; LPA, phospholipase A; LPAAT, LPA acyltransferase; MFP2, multifunctional protein; MGD, monogalactosyl diacylglycerol synthase; MGDG, monogalactosyldiacylglycerol; Mi, mitochondria; PA, phosphatidic acid; PAP1, phosphatidate phosphatase; PC, phosphatidylcholine; PE, phosphatidyl ethanolamine; PECT, ethanolamine-phosphate cytidylyltransferase; Per, peroxisome; PETA, phosphoethanolamine; PI, phosphatidylinositol; PIP, polyphosphoinositide; PIP2, phosphatidylinositol 4,5-bisphosphate; PIS1, CDP-diacylglycerol–inositol 3-phosphatidyltransferase; PLC, phospholipase C; PLDζ, phospholipase D; PS, phosphatidylserine; PSS1, CDP-diacylglycerol–serine O-phosphatidyltransferase; SQD2, sulfoquinovosyl diacylglycerol synthase; SQDG, sulfoquinovosyl diacylglycerol; TAG, triacylglycerols; TER, very-long-chain enoyl-CoA reductase; UFA, unsaturated fatty acid.

Based on the results of the transcriptomic and lipidomic analyses, a schematic diagram was proposed to illustrate the gene-metabolite network of lipids in peanut during adapting to cold stress, which clearly demonstrated the molecular regulation mechanism of membrane lipid metabolism in peanut cold tolerance. As illustrated in **Figure 8**, in membrane lipid metabolism, the increased PA induced by cold signal was immediately catalyzed by CDS1/2 and PAP1 to generate the lipid intermediates DAG and CDP-DAG, which inhibited the excessively accumulation of PA. Subsequently, the upregulation of CRLS and PIS1 accelerated the biosynthesis of CL and PI from CDP-DAG. The increased PI can not only increase the DAG level but also activated Ca^2+^ signal transduction pathway through “double messenger system”. Moreover, transcriptomic analysis showed the upregulation of SQD2, MGD, and DGD1 that can contribute to the formation of SQDG and DGDG from DAG and CDP-DAG, compensated for the decreased lipid unsaturation caused by the reduced level of MGDG. In fatty acid metabolism, the genes involved in fatty acid elongation all significantly down-regulated and the genes involved in fatty acid β-oxidation all significantly up-regulated, which prevented the formation of very long chain fatty acids (VLCFA) (i.e. fatty acids having at least 20 carbon atoms), and promoted the generation of C16 and C18 fatty acids. The increased level of C18:3 and the significant upregulation of LOX3, AOS1/3, and AOC activated the α-linolenic acid metabolism pathway, which may cause an increase in JA and activate the JA signal transduction pathway. Overall, transcriptional responses of peanut to cold stress were consistent with lipidomic changes, thus indicating the main regulations occurring at the transcriptional level.

## Discussion

Cold stress seriously affects the growth, development, yield, and quality of crops, and is a key limiting factor for peanut production at higher altitudes and colder agricultural regions (Zhang H. et al., 2019). It is well known that photosynthesis is very sensitive to cold stress (Peng et al., 2015). In this study, the similar phenomenon had been found that cold-sensitive peanut cultivars experienced pronounced decline in Pn, Fv/Fm, and Chls contents presumably caused by the damaged photosynthetic membrane. This may reduce assimilation products and cause an inhibited growth and a lower yield (Weiszmann et al., 2018). Therefore, it is considered as of the critical importance to elucidate the specific mechanism and promote the genetic improvement of cold tolerance in peanut cultivars. We studied the early changes occurring upon cold stress in the transcriptome and lipidome of peanut, which is so far the best developed gene-metabolite network for peanut adaptation to cold stress.

### Comparative Transcriptomic Analysis Revealed Lipid Metabolism May Play a Central Role in Peanut Cold Tolerance

Nowadays, thousands of genes and many signaling pathways have been identified in numerous plants during cold stress (Winfield et al., 2010), however, a clear and comprehensive relationship between lipid metabolism and cold tolerance has not been established, especially in peanuts. Recent studies suggest that plant hormone signal transduction, photosynthesis, plant-pathogen interaction, and circadian rhythms pathways were essential all play a role in response to cold stress (Li et al., 2019; Ma et al., 2019; Xin et al., 2019). In this study, these responses also were significantly enriched in two peanut cultivars under cold stress. However, lipid metabolism including fatty acid and membrane lipid metabolism pathway was the most significantly enriched in NH5 compared with FH18. It is likely that most of the cold-stress responsive genes have the similar expression patterns in both cold-tolerant and cold-sensitive varieties to cope with the cold stress. However, it is possible that all cold-responsible genes may not contribute to plant cold-tolerance. In fact, several studies have recently shown that membrane lipid remodeling can modulate the lipid composition, fatty acyl group unsaturation, and membrane fluidity, which have been developed as a key strategy for plant to cope with cold stress (Gu et al., 2017; Zuther et al., 2019).

### Upregulated Expression of *PAP1* and *CDS1* May Be Important to Prevent the Membrane Peroxidation Caused by Excessive PA Accumulation

In our study, besides the phosphorylation of DAG by DGK5 induced by the upregulated PI and PS, similar to that observed in *A. thaliana* (Hou et al., 2016), PA also largely accumulates through the *de novo* synthesis initiated by two acylations of G3P by GPAT2 and LPAAT, as well as the hydrolysis of PC and PE by PLDζs and PLDδ (**Figure 5**). However, increasing evidence suggests that the excessive PA could generate hydroperoxides and free radicals with a series of enzymes and finally lead to membrane lipid peroxidation (Li et al., 2009), which is consistent with the significantly increased EL and MDA in FH18. In the cold-tolerant cultivar NH5, two *PAP1* and one *CDS1* gene were significantly upregulated, encoding the key enzymes in the PA-DAG and PA-(CDP-DAG) pathways, respectively. The increases in DAG and CDP-DAG may activate a few key genes (*PIS1*, *CRLS*, *EPT1*, *PSS1*, *DGAT1*, *SQD2*, *MGD*, and *DGD1*) encoding lipid synthases to generate complex lipids and inhibit excessive PA accumulation, which may reduce membrane damage to a certain extent and is consistent with the stable EL and MDA content in NH5 during the early stage of cold stress.

### Elevated Levels of DGDG and SQDG Ameliorate the Photosynthetic Disorder Induced by Decreased MGDG in Peanut Under Cold Stress

The reduction in MGDG is a common plant response to osmotic stress caused by freezing, drought, or salinity (Gigon et al., 2004; Chen and Thelen, 2013; Omoto et al., 2016), which could result in serious disorder and dysfunction of photosynthetic membranes (Garab et al., 2016). While the decreased MGDG was accompanied by the increased DGDG and SQDG in NH5 because of the upregulated expression of *DGD1* and *SQD2*. In *A. thaliana*, the *dgd1* mutant hindered membrane light-harvesting complex II (LHCII)-macrodomain formation, reduced the stability of PSI, and shortened the lifetime of Chl fluorescence (Klaus et al., 2002). Therefore, the cold-induced upregulation of *DGD1* and increase in DGDG levels are important for maintaining the PSI function by protecting and stabilizing the photosynthetic apparatus in peanut. Furthermore, the increased SQDG is also required for peanut cold tolerance not only because SQDG is closely associated with the photosynthetic apparatus but also because it may play a role in signaling processes in plants. Seigneurin-Berny et al. (2000) showed that SQDG may bind annexin (cellular proteins) in a Ca^2+^-dependent manner. The family of annexins is considered to play a role in the regulation of membrane organization, membrane fusion, and ion transport across membranes.

Moreover, galactolipids are rich in unsaturated fatty acids. The extremely significant increase of C34:6-, C36:5-, and C36:6-DGDG greatly improved the lipid unsaturation in NH5 (**Figure 6A**; **Table 1**). In addition to being converted to DGDG, the rest of C36:5- and C36:6-MGDG can be also directly hydrolyzed, releasing large amounts of C18:3. The maintenance of polyunsaturated fatty acid levels in chloroplast lipids has been shown to contribute to low temperature survival and the normal formation of chloroplast membranes under cold stress (Li et al., 2015), which might also be responsible for maintaining photosynthesis.

### The Essential Role of Fatty Acid Metabolism in Peanut Cold Tolerance

Under cold stress, complex lipids can be further hydrolyzed into FFAs by PLA2 and activate fatty acid metabolic pathways such as fatty acid biosynthesis and degradation. The fatty acid elongation pathway is the key step in fatty acid biosynthesis and is mainly catalyzed by fatty acid elongase complexes (FAEs), including KCS, KCR, HACD, and TER (Haslam and Kunst, 2013). It is noteworthy that all the genes in FAE were significantly downregulated in NH5 and inhibited the elongation of fatty acids. A previous study has shown that changes in the ratio of very long chain (VLCFAs) to short chain fatty acids (SCFAs) can lead to an alteration in membrane fluidity in plants and thus the adaptation to environmental stresses (Rawsthorne, 2002). Under cold stress, the contents of UFAs and C16 and C18 fatty acids were higher, which is because C16 and C18 fatty acids have a lower melting point than C24 fatty acids, allowing membrane lipids to maintain fluidity under cold stress (Upchurch, 2008). Interestingly, KCS1 and KCS6, which are mainly responsible for the synthesis of fatty acids above 24 carbon chain length and involved in cutin, suberine, and wax biosynthesis, catalyze the first step of fatty acid elongation, and were extremely significantly downregulated in NH5 (Hooker et al., 2002). Wax is the first barrier to plant contact with the external environment and plays an important role in adaptation to sudden variations in environmental conditions (Xue et al., 2017; He et al., 2019). The relationship between wax secretion and peanut cold tolerance deserves further study.

Fatty acid β-oxidation is the main form of fatty acid degradation in plants and is catalyzed by a multienzyme complex including ACOX, multifunctional protein (MFP), and ACAA to decrease the carbon chain length of fatty acids (Yusupov et al., 2010). In the present study, *ACOX1* and *ACAA1* were significantly upregulated in NH5 and activated the β-oxidation pathway of fatty acids under cold stress. Fatty acid β-oxidation is the sole pathway for metabolic breakdown of fatty acids to generate energy and carbon skeletons in plants and plays an important role in plant growth, development and cellular homeostasis (Arent et al., 2010; Jiang et al., 2011). Moreover, fatty acid β-oxidation is also the central part of α-linolenic acid (C18:3) metabolism. Under cold stress, the large amount of accumulated C18:3 caused by the hydrolyzation of C36:5- and C36:6-MGDG in NH5 was sequentially metabolized by LOX, AOS, and AOC into 12-oxo-phytodienoic acid (OPDA) or dinor-OPDA (dnOPDA) and finally underwent three cycles of β-oxidation to yield JA. In *A. thaliana*, JA positively modulates the CBF pathway, leading to the accumulation of cryoprotective compounds and interacting with plant phytohormones to regulate stomatal closure and maintain photosynthesis under cold stress (Verma et al., 2016; Hu et al., 2017). Therefore, we conclude that fatty acid β-oxidation and JA biosynthesis may directly improve peanut cold tolerance through CBF-dependent signaling and plant hormone signal transduction pathways.

In conclusion, the present results revealed that, under cold stress, the cytoplasmic membrane and organellar membrane of peanut plants were severely damaged, the photosynthetic capacity decreased significantly, and the plant growth was inhibited. During the adaptation of peanut plants to cold stress, lipid metabolism including membrane lipid and fatty acid metabolism was the significant contributor. The phospholipid synthesis pathway in ER, and the galactolipid synthesis pathway and the α-linolenic acid metabolism pathway in chloroplast were activated, besides, most of the genes that catalyze these reactions were up-regulated. The upregulation of *PAP1* and *CDS1/2* under cold stress can inhibit the damage of membrane lipid peroxidation caused by excessive accumulation of PA. The upregulation of *MGD*, *DGD1*, and *SQD2* caused the increase of DGDG and SQDG content, which was crucial to the maintenance of chloroplast structural integrity and normal photosynthesis. α-Linolenic acid metabolism and fatty acid β-oxidation may improve peanut cold tolerance by partially modulating the JA signal transduction pathway. This study lays the foundation for deeply analyzing the molecular mechanism and realizing the genetic improvement of peanut cold tolerance.

## Data Availability Statement

The datasets generated for this study can be found in the SRA accession number: PRJNA602777.

## Author Contributions

HZ designed and performed the whole experiment. CJ, JR, and JD identified the cold-tolerant and cold-sensitive peanut varieties and measured the morphological indexes. XS and XZ helped to measure physiological indicators. XW, JW, and CZ helped to conduct the qRT-PCR test. SZ, XL, and SG assisted in data analysis. HY revised the manuscript.

## Conflict of Interest

The authors declare that the research was conducted in the absence of any commercial or financial relationships that could be construed as a potential conflict of interest.

## References

[B1] Almadanim M. C. Alexandre B. M. Rosa M. T. G. Sapeta H. Leitao A. E. Ramalho J. C. (2017). Rice calcium-dependent protein kinase OsCPK17 targets plasma membrane intrinsic protein and sucrose-phosphate synthase and is required for a proper cold stress response. Plant Cell Environ. 40, 1197–1213. 10.1111/pce.12916 28102545

[B2] Arent S. Christensen C. E. Pye V. E. Norgaard A. Henriksen A. (2010). The multifunctional protein in peroxisomal beta-oxidation: structure and substrate specificity of the *Arabidopsis thaliana* protein MFP2. J. Biol. Chem. 285, 24066–24077. 10.1074/jbc.M110.106005 20463021 PMC2911295

[B3] Arisz S. A. Wijk R. V. Roels W. Zhu J. K. Haring M. A. Munnik T. (2013). Rapid phosphatidic acid accumulation in response to low temperature stress in Arabidopsis is generated through diacylglycerol kinase. Front. Plant Sci. 4, 1. 10.3389/fpls.2013.00001 23346092 PMC3551192

[B4] Bai H. Zhang Y. Yu H. Irfan M. Huang Y. Han M. (2019). Phylogenetic diversity and cross-inoculation of indigenous isolated Bradyrhizobium from nodules of peanut in Liaoning province of China. Mol. Biol. Res. Commun. 8, 59–68. 10.22099/mbrc.2019.32983.1392 31531377 PMC6715265

[B5] Barrero-Sicilia C. Silvestre S. Haslam R. P. Michaelson L. V. (2017). Lipid remodelling: Unravelling the response to cold stress in Arabidopsis and its extremophile relative Eutrema salsugineum. Plant Sci. 263, 194–200. 10.1016/j.plantsci.2017.07.017 28818375 PMC5567406

[B6] Cantrel C. Vazquez T. Puyaubert J. Reze N. Lesch M. Kaiser W. M. (2011). Nitric oxide participates in cold-responsive phosphosphingolipid formation and gene expression in Arabidopsis thaliana. New Phytol. 189, 415–427. 10.1111/j.1469-8137.2010.03500.x 21039566

[B7] Chang B. W. Zhong P. Liu J. Tang Z. H. Gao Y. B. Yu H. J. (2019). Effect of low-temperature stress and gibberellin on seed germination and seedling physiological responses in peanut. Acta Agronomica Sin. 45, 118–130. 10.3724/SP.J.1006.2019.84043

[B8] Chen M. Thelen J. J. (2013). ACYL-LIPID DESATURASE2 is required for chilling and freezing tolerance in Arabidopsis. Plant Cell 25, 1430–1444. 10.1105/tpc.113.111179 23585650 PMC3663278

[B9] Chen N. Yang Q. L. Hu D. Q. Pan L. J. Chi X. Y. Chen M. N. (2014). Gene expression profiling and identification of resistance genes to low temperature in leaves of peanut (Arachis hypogaea L.). Sci. Hortic. 169, 214–225. 10.1016/j.scienta.2014.01.043

[B10] Chen X. Song F. Liu F. Tian X. Liu S. Xu H. (2014). Effect of different arbuscular mycorrhizal fungi on growth and physiology of maize at ambient and low temperature regimes. Sci. World J. 2014, 956141. 10.1155/2014/956141 PMC4032736 24895680

[B11] Ding Y. Li H. Zhang X. Xie Q. Gong Z. Yang S. (2015). OST1 kinase modulates freezing tolerance by enhancing ICE1 stability in Arabidopsis. Dev. Cell 32, 278–289. 10.1016/j.devcel.2014.12.023 25669882

[B12] Dubots E. Botte C. Boudiere L. Yamaryo B. Y. Jouhet J. Marechal E. (2012). Role of phosphatidic acid in plant galactolipid synthesis. Biochimie 94, 86–93. 10.1016/j.biochi.2011.03.012 21501653

[B13] Fan J. Yu L. Xu C. (2017). A central role for triacylglycerol in membrane lipid breakdown, fatty acid beta-oxidation, and plant survival under extended darkness. Plant Physiol. 174, 1517–1530. 10.1104/pp.17.00653 28572457 PMC5490926

[B14] Garab G. Ughy B. Goss R. (2016). Role of MGDG and non-bilayer lipid phases in the structure and dynamics of chloroplast thylakoid membranes. Subcell. Biochem. 86, 127–157. 10.1007/978-3-319-25979-6_6 27023234

[B15] Gigon A. Matos A. R. Laffray D. Zuily-Fodil Y. Pham-Thi A. T. (2004). Effect of drought stress on lipid metabolism in the leaves of *Arabidopsis thaliana* (ecotype Columbia). Ann. Bot. 94, 345–351. 10.1093/aob/mch150 15277243 PMC4242175

[B16] Gu Y. He L. Zhao C. Wang F. Yan B. Gao Y. (2017). Biochemical and transcriptional regulation of membrane lipid metabolism in maize leaves under low temperature. Front. Plant Sci. 8, 2053. 10.3389/fpls.2017.02053 29250095 PMC5714865

[B17] Gwak Y. Hwang Y. S. Wang B. Kim M. Jeong J. Lee C. G. (2014). Comparative analyses of lipidomes and transcriptomes reveal a concerted action of multiple defensive systems against photooxidative stress in Haematococcus pluvialis. J. Exp. Bot. 65, 4317–4334. 10.1093/jxb/eru206 24821952 PMC4112636

[B18] Han Q. H. Huang B. Ding C. B. Zhang Z. W. Chen Y. E. Hu C. (2017). Effects of melatonin on anti-oxidative systems and photosystem II in cold-stressed rice seedlings. Front. Plant Sci. 8, 785. 10.3389/fpls.2017.00785 28553310 PMC5425610

[B19] Hantzis L. J. Kroh G. E. Jahn C. E. Cantrell M. Peers G. Pilon M. (2018). A program for iron economy during deficiency targets specific Fe proteins. Plant Physiol. 176, 596–610. 10.1104/pp.17.01497 29150559 PMC5761800

[B20] Haslam T. M. Kunst L. (2013). Extending the story of very-long-chain fatty acid elongation. Plant Sci. 210, 93–107. 10.1016/j.plantsci.2013.05.008 23849117

[B21] He J. Tang S. Yang D. Chen Y. Ling L. Zou Y. (2019). Chemical and transcriptomic analysis of cuticle lipids under cold Stress in *Thellungiella salsuginea*. Int. J. Mol. Sci. 20, 4519. 10.3390/ijms20184519 PMC6770325 31547275

[B22] Hooker T. S. Millar A. A. Kunst L. (2002). Significance of the expression of the CER6 condensing enzyme for cuticular wax production in Arabidopsis. Plant Physiol. 129, 1568–1580. 10.1104/pp.003707 12177469 PMC166744

[B23] Hou Q. Ufer G. Bartels D. (2016). Lipid signalling in plant responses to abiotic stress. Plant Cell Environ. 39, 1029–1048. 10.1111/pce.12666 26510494

[B24] Hu Y. Jiang Y. Han X. Wang H. Pan J. Yu D. (2017). Jasmonate regulates leaf senescence and tolerance to cold stress: crosstalk with other phytohormones. J. Exp. Bot. 68, 1361–1369. 10.1093/jxb/erx004 28201612

[B25] Huang B. Qi F. Sun Z. Miao L. Zhang Z. Liu H. (2019). Marker-assisted backcrossing to improve seed oleic acid content in four elite and popular peanut (*Arachis hypogaea* L.) cultivars with high oil content. Breed. Sci. 69, 234–243. 10.1270/jsbbs.18107 31481832 PMC6711728

[B26] Jiang T. Zhang X. F. Wang X. F. Zhang D. P. (2011). Arabidopsis 3-ketoacyl-CoA thiolase-2 (KAT2), an enzyme of fatty acid beta-oxidation, is involved in ABA signal transduction. Plant Cell Physiol. 52, 528–538. 10.1093/pcp/pcr008 21257607

[B27] Karami-Moalem S. Maali-Amiri R. Kazemi-Shahandashti S. S. (2018). Effect of cold stress on oxidative damage and mitochondrial respiratory properties in chickpea. Plant Physiol. Biochem. 122, 31–39. 10.1016/j.plaphy.2017.11.011 29172103

[B28] Kim K. N. Cheong Y. H. Grant J. J. Pandey G. K. Luan S. (2003). CIPK3, a calcium sensor-associated protein kinase that regulates abscisic acid and cold signal transduction in Arabidopsis. Plant Cell 15, 411–423. 10.1105/tpc.006858 12566581 PMC141210

[B29] Klaus D. Hartel H. Fitzpatrick L. M. Froehlich J. E. Hubert J. Benning C. (2002). Digalactosyldiacylglycerol synthesis in chloroplasts of the Arabidopsis *dgd1* mutant. Plant Physiol. 128, 885–895. 10.1104/pp.010780 11891245 PMC152202

[B30] Li W. Wang R. Li M. Li L. Wang C. Welti R. (2008). Differential degradation of extraplastidic and plastidic lipids during freezing and post-freezing recovery in *Arabidopsis thaliana*. J. Biol. Chem. 283, 461–468. 10.1074/jbc.M706692200 17962199

[B31] Li M. Hong Y. Wang X. (2009). Phospholipase D- and phosphatidic acid-mediated signaling in plants. Biochim. Biophys. Acta 1791, 927–935. 10.1016/j.bbalip.2009.02.017 19289179

[B32] Li Q. Zheng Q. Shen W. Cram D. Fowler D. B. Wei Y. (2015). Understanding the biochemical basis of temperature-induced lipid pathway adjustments in plants. Plant Cell 27, 86–103. 10.1105/tpc.114.134338 25564555 PMC4330585

[B33] Li Y. Wang X. Ban Q. Zhu X. Jiang C. Wei C. (2019). Comparative transcriptomic analysis reveals gene expression associated with cold adaptation in the tea plant Camellia sinensis. BMC Genomics 20, 624. 10.1186/s12864-019-5988-3 31366321 PMC6670155

[B34] Lichtenthaler H. K. Wellburn A. (1983). Determinations of total carotenoids and chlorophylls a and b of leaf extracts in different solvents. Biochem. Soc T. 11, 591–592. 10.1042/bst0110591

[B35] Ma Y. Dai X. Xu Y. Luo W. Zheng X. Zeng D. (2015). COLD1 confers chilling tolerance in rice. Cell 160, 1209–1221. 10.1016/j.cell.2015.01.046 25728666

[B36] Ma L. Coulter J. A. Liu L. Zhao Y. Chang Y. Pu Y. (2019). Transcriptome analysis reveals key cold-stress-responsive genes in winter rapeseed (*Brassica rapa* L.). Int. J. Mol. Sci. 20, 1071. 10.3390/ijms20051071 PMC6429191 30832221

[B37] Narayanan S. Prasad P. V. Welti R. (2016). Wheat leaf lipids during heat stress: II. Lipids experiencing coordinated metabolism are detected by analysis of lipid co-occurrence. Plant Cell Environ. 39, 608–617. 10.1111/pce.12648 26436445 PMC5141584

[B38] Noblet A. Leymarie J. Bailly C. (2017). Chilling temperature remodels phospholipidome of Zea mays seeds during imbibition. Sci. Rep. 7, 8886. 10.1038/s41598-017-08904-z 28827663 PMC5566375

[B39] Omoto E. Iwasaki Y. Miyake H. Taniguchi M. (2016). Salinity induces membrane structure and lipid changes in maize mesophyll and bundle sheath chloroplasts. Physiol. Plant 157, 13–23. 10.1111/ppl.12404 26555406

[B40] Peng X. Teng L. Yan X. Zhao M. Shen S. (2015). The cold responsive mechanism of the paper mulberry: decreased photosynthesis capacity and increased starch accumulation. BMC Genomics 16, 898. 10.1186/s12864-015-2047-6 26537770 PMC4634900

[B41] Peppino M. M. Reyna M. Meringer M. V. Racagni G. E. Villasuso A. L. (2017). Lipid signalling mediated by PLD/PA modulates proline and H_2_O_2_ levels in barley seedlings exposed to short- and long-term chilling stress. Plant Physiol. Biochem. 113, 149–160. 10.1016/j.plaphy.2017.02.008 28214728

[B42] Rawsthorne S. (2002). Carbon flux and fatty acid synthesis in plants. Prog. Lipid Res. 41, 182–196. 10.1016/S0163-7827(01)00023-6 11755683

[B43] Seigneurin-Berny D. Rolland N. Dorne A. J. Joyard J. (2000). Sulfolipid is a potential candidate for annexin binding to the outer surface of chloroplast. Biochem. Biophys. Res. Commun. 272, 519–524. 10.1006/bbrc.2000.2805 10833445

[B44] Sui N. Wang Y. Liu S. Yang Z. Wang F. Wan S. (2018). Transcriptomic and physiological evidence for the relationship between unsaturated fatty acid and salt stress in peanut. Front. Plant Sci. 9, 7. 10.3389/fpls.2018.00007 29403517 PMC5786550

[B45] Suzuki S. Awai K. Ishihara A. Yamauchi K. (2016). Cold temperature blocks thyroid hormone-induced changes in lipid and energy metabolism in the liver of Lithobates catesbeianus tadpoles. Cell Biosci. 6, 19. 10.1186/s13578-016-0087-5 26981232 PMC4792105

[B46] Tan W. J. Yang Y. C. Zhou Y. Huang L. P. Xu L. Chen Q. F. (2018). DIACYLGLYCEROL ACYLTRANSFERASE and DIACYLGLYCEROL KINASE modulate triacylglycerol and phosphatidic acid production in the plant response to freezing stress. Plant Physiol. 177, 1303–1318. 10.1104/pp.18.00402 29853600 PMC6053003

[B47] Upchurch R. G. (2008). Fatty acid unsaturation, mobilization, and regulation in the response of plants to stress. Biotechnol. Lett. 30, 967–977. 10.1007/s10529-008-9639-z 18227974

[B48] Verma V. Ravindran P. Kumar P. P. (2016). Plant hormone-mediated regulation of stress responses. BMC Plant Biol. 16, 86. 10.1186/s12870-016-0771-y 27079791 PMC4831116

[B49] Vincent M. V. Zhang J. Guan S. Y. Thobela L. T. Wang P. W. Wang X. Z. (2018). Evaluation of groundnut (*Arachis hypogaea* L.) germplasm for chilling tolerance utilizing physiological and biochemical parameters. J. Peanut Sci. 47, 13–23.

[B50] Weiszmann J. Furtauer L. Weckwerth W. Nagele T. (2018). Vacuolar sucrose cleavage prevents limitation of cytosolic carbohydrate metabolism and stabilizes photosynthesis under abiotic stress. FEBS J. 285, 4082–4098. 10.1111/febs.14656 30216682

[B51] Winfield M. O. Lu C. Wilson I. D. Coghill J. A. Edwards K. J. (2010). Plant responses to cold: Transcriptome analysis of wheat. Plant Biotechnol. J. 8, 849–871. 10.1111/j.1467-7652.2010.00536.x 20561247

[B52] Xiao F. Song L. (2011). Analysis of extreme low-temperature events during the warm season in Northeast China. Nat. Hazards 58, 1333–1344. 10.1007/s11069-011-9735-6

[B53] Xin H. Xianchao N. Pan X. Wei L. Min Y. Yu K. (2019). Comparative transcriptome analyses revealed conserved and novel responses to cold and freezing stress in *Brassica napus* L. G3 (Bethesda) 9, 2723–2737. 10.1534/g3.119.400229 31167831 PMC6686917

[B54] Xue D. Zhang X. Lu X. Chen G. Chen Z. H. (2017). Molecular and evolutionary mechanisms of cuticular wax for plant drought tolerance. Front. Plant Sci. 8, 621. 10.3389/fpls.2017.00621 28503179 PMC5408081

[B55] Yuan P. Yang T. Poovaiah B. W. (2018). Calcium signaling-mediated plant response to cold stress. Int. J. Mol. Sci. 19, e3896. 10.3390/ijms19123896 30563125 PMC6320992

[B56] Yusupov R. Finegold D. N. Naylor E. W. Sahai I. Waisbren S. Levy H. L. (2010). Sudden death in medium chain acyl-coenzyme a dehydrogenase deficiency (MCADD) despite newborn screening. Mol. Genet. Metab. 101, 33–39. 10.1016/j.ymgme.2010.05.007 20580581

[B57] Zhang G. H. Yu S. T. Wang H. Wang X. D. (2019). Transcriptome profiling of high oleic peanut under low temperature during germination. Yi Chuan 41, 1050–1059. 10.16288/j.yczz.19-097 31735707

[B58] Zhang H. Dong J. Zhao X. Zhang Y. Ren J. Xing L. (2019). Research progress in membrane lipid metabolism and molecular mechanism in peanut cold tolerance. Front. Plant Sci. 10, 838. 10.3389/fpls.2019.00838 31316538 PMC6610330

[B59] Zuther E. Schaarschmidt S. Fischer A. Erban A. Pagter M. Mubeen U. (2019). Molecular signatures associated with increased freezing tolerance due to low temperature memory in Arabidopsis. Plant Cell Environ. 42, 854–873. 10.1111/pce.13502 30548618

