# Supplementary material for: An Advanced Lipid Metabolism System Revealed by Transcriptomic and Lipidomic Analyses Plays a Central Role in Peanut Cold Tolerance
Source: Front Plant Sci. 2020 Jul 21;11:1110. doi: 10.3389/fpls.2020.01110 (PMC7396583; doi:10.3389/fpls.2020.01110)
Supplement: Supplementary file 1 [file DataSheet_1.zip › Supplementary Material/Figures.pdf]

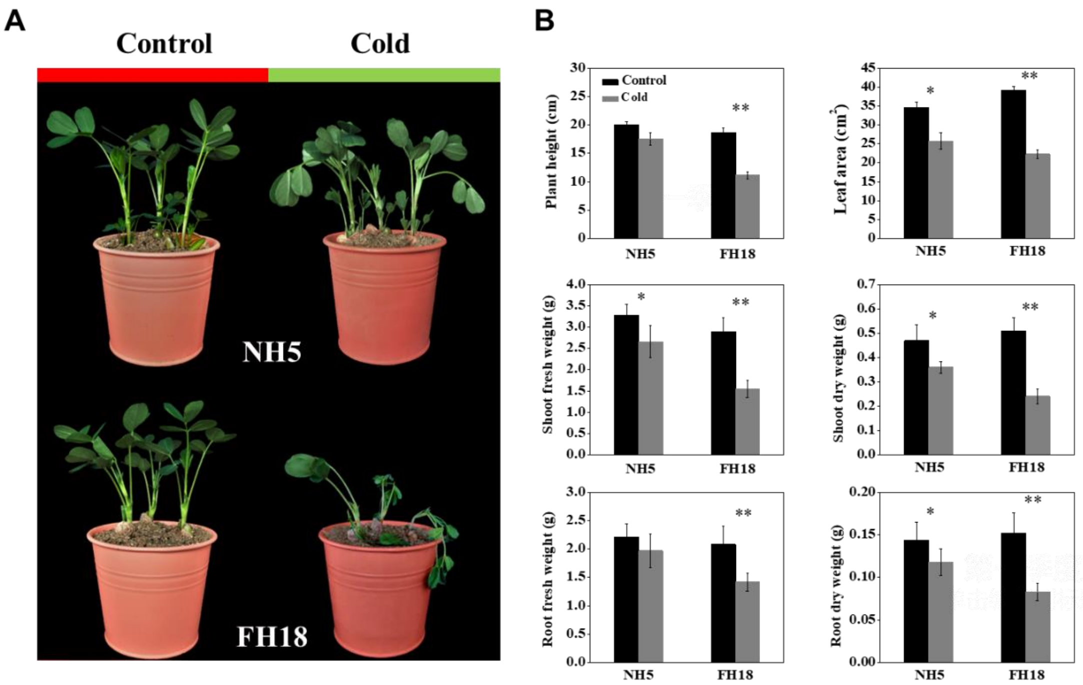

Figure S1. The changes of phenotype and morphology before and after cold treatment in NH5 and FH18. (A) Phenotypes of NH5 and FH18 under control and cold conditions respectively. (B) Morphological indexes of NH5 and FH18 under control and cold conditions including plant height, leaf area, shoot fresh weight, shoot dry weight, root fresh weight and root dry weight. Error bars represent the SD of the means (n=3). *P < 0.05; **P < 0.01.

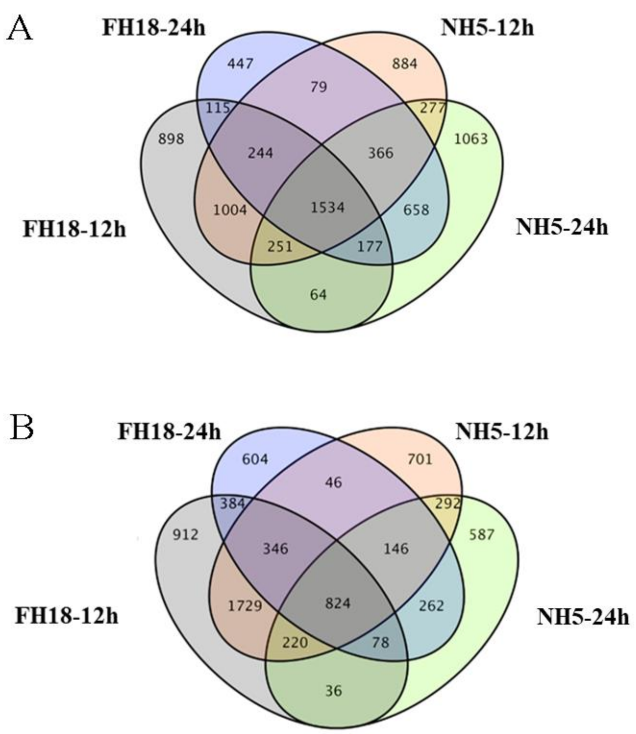

Figure S2. The number of continuously differentially expressed genes (CDEGs) in NH5 and FH18 during the early stage of cold stress. (A) the up-regulated CDEGs in every sample. (B) the down-regulated CDEGs in every sample.
