# Supplementary material for: An Advanced Lipid Metabolism System Revealed by Transcriptomic and Lipidomic Analyses Plays a Central Role in Peanut Cold Tolerance
Source: Front Plant Sci. 2020 Jul 21;11:1110. doi: 10.3389/fpls.2020.01110 (PMC7396583; doi:10.3389/fpls.2020.01110)
Supplement: Supplementary file 1 [file DataSheet_1.zip › Supplementary Material/Method S1.docx]

**Method S1** Experiment method of Lipid profiling by ESI triple–quadrupole MS

Total lipids were dissolved in 1 mL chloroform/methanol (1:1, v/v). The lipids in each class were quantified in comparison to two internal standards of the class. For each analytical sample, internal standards in 20 µL and a volume of sample corresponding to 0.04 mg extracted leaf dry mass were added to a 2 mL of vial. Then, samples were brought to 1.4 mL by adding chloroform: methanol: 300 mM ammonium acetate in water (30/66.5/3.5, v/v/v) for mass spectrometric analysis. Internal standards include 0.15 nmol 14:0/14:0-PA, 0.15 nmol 20:0/20:0 (phytanoyl)-PA, 0.30 nmol 12:0/12:0-PC, 0.30 nmol 24:1/24:1-PC, 0.15 nmol 12:0/12:0-PE, 0.15 nmol 20:0/20:0 (phytanoyl)-PE, 0.15 nmol 14:0/14:0-PG, 0.15 nmol 20:0/20:0 (phytanoyl)-PG, 0.14 nmol 16:0/18:0-PI, 0.05 nmol 18:0/18:0-PI, 0.10 nmol 14:0/14:0-PS, 0.10 nmol 20:0/20:0 (phytanoyl)-PS, 0.30 nmol 13:0-LPC, 0.30 nmol 19:0-LPC, 0.15 nmol 14:0-LPE, 0.15 nmol 18:0-LPE, 0.15 nmol 14:0-LPG, 0.15 nmol 18:0-LPG, 1.50 nmol 18:0/16:0-MGDG, 1.30 nmol 18:0/18:0-MGDG, 0.35 nmol 18:0/16:0-DGDG, 0.95 nmol 18:0/18:0-DGDG, 1.55 nmol 17:1/17:1/17:1-TAG. Data were acquired by an Agilent Technologies 6460 triple quadruple electrospray ionization mass spectrometer (ESI/MS) (Santa Clara, CA, USA) with a multiple reaction monitoring method, operating in direct infusion mode. The source temperature (heated nebulizer) was 100°C, and -4.5 or +5.5 kV was applied to the electrospray capillary. The two ion source gases were each set at 45 arbitrary units; the curtain gas was set at 20 arbitrary units. The entrance potential was ±10 to ±15 V. 400 μL from each sample were used to fill a 300-μl loop for infusion at 30 μL min^−1^. Infusion was performed twice, acquiring data in positive and negative modes separately. The collision energies, with nitrogen in the collision cell, were 17 V for PA, 28 V for PC, 20 V for PE, 17 V for PG, 17 V for PS, 39 V for PI, 28 V for LPC, 20 V for LPE, 17 V for LPG, 14 V for MGDG, 50 V for DGDG, 23 V for TAG. The mass analyzers were adjusted to a resolution of 0.7 amu full width at half height. For each spectrum, 9–150 continuum scans were averaged in multiple channel analyzer mode.
