# Supplementary material for: An Advanced Lipid Metabolism System Revealed by Transcriptomic and Lipidomic Analyses Plays a Central Role in Peanut Cold Tolerance
Source: Front Plant Sci. 2020 Jul 21;11:1110. doi: 10.3389/fpls.2020.01110 (PMC7396583; doi:10.3389/fpls.2020.01110)
Supplement: Supplementary file 1 [file DataSheet_1.zip › Supplementary Material/Table S15.docx]

**Table S15.** The list of 59 lipid-related continuously differentially expressed genes (CDEGs)

| **Tifrunner.gnm1. ann1. CCJH** | **Gene name** | **Putative function** | **log2FC (T1/T0)** | **log2FC (T2/T0)** |
| --- | --- | --- | --- | --- |
| **DAG/TAG *de novo* synthesis** | |  |  |  |
| arahy.Tifrunner.gnm1.ann1.5W0QMQ | DGAT1 | diacylglycerol acyltransferase 1 | 1.79 | 3.02 |
| arahy.Tifrunner.gnm1.ann1.5U50HE | GPAT2 | glycerol-3-phosphate acyltransferase | 9.10 | 10.31 |
| arahy.Tifrunner.gnm1.ann1.K79JM6 | GPAT2 | glycerol-3-phosphate acyltransferase | 7.96 | 9.03 |
| arahy.Tifrunner.gnm1.ann1.9A7I0S | PAP1 | phosphatidate phosphatase PAH1/LPIN | 2.83 | 3.40 |
| arahy.Tifrunner.gnm1.ann1.53JHUG | PAP1 | phosphatidate phosphatase PAH1/LPIN | 2.49 | 3.08 |
| arahy.Tifrunner.gnm1.ann1.E4P4QB | PLA2/LPAAT | phospholipase A2 / LPA acyltransferase | 2.37 | 3.36 |
| arahy.Tifrunner.gnm1.ann1.ZN271A | PLA2/LPAAT | phospholipase A2 / LPA acyltransferase | 1.17 | 1.95 |
| **Membrane lipids metabolism** | |  |  |  |
| arahy.Tifrunner.gnm1.ann1.YI6E2M | AKR1B | aldehyde reductase | -2.72 | -3.29 |
| arahy.Tifrunner.gnm1.ann1.5320XR | CDS1/2 | phosphatidate cytidylyltransferase | 1.42 | 1.49 |
| arahy.Tifrunner.gnm1.ann1.GT1I17 | CK1/EK1 | choline/ethanolamine kinase | 3.13 | 4.59 |
| arahy.Tifrunner.gnm1.ann1.JJP1G0 | CRLS/CLS | cardiolipin synthase | 2.27 | 2.01 |
| arahy.Tifrunner.gnm1.ann1.KAI8DY | CRLS/CLS | cardiolipin synthase | 1.35 | 1.53 |
| arahy.Tifrunner.gnm1.ann1.BQ87IR | DGD1 | digalactosyldiacylglycerol synthase 1 | 2.71 | 2.98 |
| arahy.Tifrunner.gnm1.ann1.G7M3GH | DGK5 | diacylglycerol kinase 5 | 3.83 | 4.63 |
| arahy.Tifrunner.gnm1.ann1.UA9SQ6 | EPT1 | ethanolaminephosphotransferase | 1.06 | 2.23 |
| arahy.Tifrunner.gnm1.ann1.REV2BJ | EPT1 | ethanolaminephosphotransferase | 1.25 | 1.52 |
| arahy.Tifrunner.gnm1.ann1.8WED6E | ETNK | ethanolamine kinase | 5.10 | 3.80 |
| arahy.Tifrunner.gnm1.ann1.V1ADX0 | GLA | alpha-galactosidase | -2.58 | -1.85 |
| arahy.Tifrunner.gnm1.ann1.US8HBC | GLA | alpha-galactosidase | -2.81 | -2.56 |
| arahy.Tifrunner.gnm1.ann1.67NNK3 | MGD | 1,2-diacylglycerol 3-beta-galactosyltransferase | 1.36 | 2.09 |
| arahy.Tifrunner.gnm1.ann1.HAI9AH | MGD | 1,2-diacylglycerol 3-beta-galactosyltransferase | 1.02 | 1.72 |
| arahy.Tifrunner.gnm1.ann1.YU79V3 | PECT/ET | ethanolamine-phosphate cytidylyltransferase | 1.33 | 1.74 |
| arahy.Tifrunner.gnm1.ann1.9Q6XN1 | PECT/ET | ethanolamine-phosphate cytidylyltransferase | 1.07 | 1.35 |
| arahy.Tifrunner.gnm1.ann1.U045QQ | PIS1 | CDP-diacylglycerol--inositol 3-phosphatidyltransferase | -1.99 | -2.10 |
| arahy.Tifrunner.gnm1.ann1.XZ9AUI | PLDζ | phospholipase D Z | 2.99 | 4.80 |
| arahy.Tifrunner.gnm1.ann1.T1UA0C | PLDζ1 | phospholipase D zeta 1 | 3.60 | 3.75 |
| arahy.Tifrunner.gnm1.ann1.Y1617F | PLDζ2 | phospholipase D zeta 2 | 5.48 | 5.24 |
| arahy.Tifrunner.gnm1.ann1.8F881C | PSS1 | CDP-diacylglycerol--serine O-phosphatidyltransferase 1 | 1.32 | 1.96 |
| arahy.Tifrunner.gnm1.ann1.A779NY | PSS1 | CDP-diacylglycerol--serine O-phosphatidyltransferase 1 | 1.03 | 1.68 |
| arahy.Tifrunner.gnm1.ann1.IP4RNA | SPHK1 | sphingosine kinase | 1.26 | 1.96 |
| arahy.Tifrunner.gnm1.ann1.YND711 | SPHK1 | sphingosine kinase | 1.49 | 1.26 |
| arahy.Tifrunner.gnm1.ann1.L6AUEE | SQD2 | sulfoquinovosyltransferase | 1.41 | 1.73 |
| **fatty acid metabolism pathway** | |  |  |  |
| arahy.Tifrunner.gnm1.ann1.K55J2B | ACAA1 | acetyl-CoA acyltransferase 1 | 2.05 | 2.40 |
| arahy.Tifrunner.gnm1.ann1.74QZJ8 | ACOT1/2/4 | acyl-coenzyme A thioesterase 1/2/4 | 3.51 | 4.52 |
| arahy.Tifrunner.gnm1.ann1.I9A8PV | ACOX1 | acyl-CoA oxidase | 3.36 | 2.24 |
| arahy.Tifrunner.gnm1.ann1.H5H05M | AOC | allene oxide cyclase | 4.87 | 2.06 |
| arahy.Tifrunner.gnm1.ann1.F73TU2 | AOS3 | allene oxide synthase 3 | 4.12 | 1.70 |
| **Tifrunner.gnm1. ann1. CCJH** | **Gene name** | **Putative function** | **log2FC (T1/T0)** | **log2FC (T2/T0)** |
| arahy.Tifrunner.gnm1.ann1.I2ECPT | AOS1 | allene oxide synthase 1 | 4.19 | 1.98 |
| arahy.Tifrunner.gnm1.ann1.B9A644 | AOS1 | allene oxide synthase 1 | 4.19 | 1.98 |
| arahy.Tifrunner.gnm1.ann1.DB4GMR | AOS3 | allene oxide synthase 1 | 3.30 | 1.52 |
| arahy.Tifrunner.gnm1.ann1.EK4G75 | JMT | jasmonate O-methyltransferase | 13.17 | 11.53 |
| arahy.Tifrunner.gnm1.ann1.PVQF0C | JMT | jasmonate O-methyltransferase | 11.40 | 9.62 |
| arahy.Tifrunner.gnm1.ann1.4PU0PP | HACD | (3R)-3-hydroxyacyl-CoA dehydratase | -1.54 | -1.43 |
| arahy.Tifrunner.gnm1.ann1.AAI0ZL | JMT | jasmonate O-methyltransferase | 7.59 | 8.80 |
| arahy.Tifrunner.gnm1.ann1.YC7CVR | JMT | jasmonate O-methyltransferase | 7.92 | 7.26 |
| arahy.Tifrunner.gnm1.ann1.0GWZ16 | KCR1 | very-long-chain 3-oxoacyl-CoA reductase 1 | -1.27 | -1.44 |
| arahy.Tifrunner.gnm1.ann1.KTR7HF | KCR1 | very-long-chain 3-oxoacyl-CoA reductase 1 | -1.50 | -2.09 |
| arahy.Tifrunner.gnm1.ann1.AYAJ7Z | KCS1 | 3-ketoacyl-CoA synthase 1 | -4.64 | -4.87 |
| Arachis_hypogaea_newGene_17195 | KCS6 | 3-ketoacyl-CoA synthase 6 | -11.41 | -10.74 |
| arahy.Tifrunner.gnm1.ann1.6TJ5BP | LOX3 | lipoxygenase/linoleate 13S-lipoxygenase 3-1 | 2.88 | 4.83 |
| arahy.Tifrunner.gnm1.ann1.S6TVHJ | LOX3 | lipoxygenase 3 | 2.48 | 4.09 |
| arahy.Tifrunner.gnm1.ann1.7CJ2RM | LOX3 | lipoxygenase 3 | 1.92 | 3.64 |
| arahy.Tifrunner.gnm1.ann1.CU7JXX | LOX3 | lipoxygenase 3 | 1.53 | 1.86 |
| arahy.Tifrunner.gnm1.ann1.ZS7YSR | LOX5 | linoleate 9S-lipoxygenase 5 | 3.76 | 6.67 |
| arahy.Tifrunner.gnm1.ann1.V7KTJ4 | LOX5 | linoleate 9S-lipoxygenase 5 | 2.75 | 1.65 |
| arahy.Tifrunner.gnm1.ann1.ZR4S3S | MGLL/CSE | acylglycerol lipase/caffeoylshikimate esterase | 1.21 | 1.87 |
| arahy.Tifrunner.gnm1.ann1.FWY8EA | MGLL/CSE | acylglycerol lipase/caffeoylshikimate esterase | 1.43 | 1.81 |
| arahy.Tifrunner.gnm1.ann1.5S3M3D | TER | very-long-chain enoyl-CoA reductase | -2.43 | -3.10 |
| arahy.Tifrunner.gnm1.ann1.4ZRU7U | TER | very-long-chain enoyl-CoA reductase | -2.91 | -3.54 |
