# Supplementary material for: An Advanced Lipid Metabolism System Revealed by Transcriptomic and Lipidomic Analyses Plays a Central Role in Peanut Cold Tolerance
Source: Front Plant Sci. 2020 Jul 21;11:1110. doi: 10.3389/fpls.2020.01110 (PMC7396583; doi:10.3389/fpls.2020.01110)
Supplement: Supplementary file 1 [file DataSheet_1.zip › Supplementary Material/Table S2.docx]

**Table S2**. The changes of chlorophyll content before and after cold treatments in NH5 and FH18

| **Treat time** | **Chl-a (mg g^-1^ FW)** | | **Chl-b (mg g^-1^ FW)** | | **Chl-a/b** | | **Chl-total (mg g^-1^ FW)** | |
| --- | --- | --- | --- | --- | --- | --- | --- | --- |
|  | **NH5** | **FH18** | **NH5** | **FH18** | **NH5** | **FH18** | **NH5** | **FH18** |
| 0h | 3.30±0.02^a^ | 3.36±0.11^a^ | 0.68±0.01^a^ | 0.57±0.10^a^ | 4.85±0.04^a^ | 5.93±0.10^a^ | 3.98±0.02^a^ | 3.92±0.09B^a^ |
| 12h | 2.90±0.06^ab^ | 2.43±0.07^b^ | 0.65±0.08^a^ | 0.50±0.02^b^ | 4.48±0.10^ab^ | 4.87±0.04^b^ | 3.55±0.10^ab^ | 2.93±0.05^b^ |
| 24h | 2.68±0.09^ab^ | 1.71±0.05^c^ | 0.62±0.03^a^ | 0.45±0.03^bc^ | 4.30±0.06^ab^ | 3.83±0.03^c^ | 3.31±0.08^ab^ | 2.16±0.07^c^ |
| 48h | 2.32±0.17^b^ | 1.26±0.10^d^ | 0.59±0.03^a^ | 0.39±0.02^c^ | 3.91±0.02^b^ | 3.23±0.10^d^ | 2.91±0.20^b^ | 1.65±0.09^d^ |
| 72h | 1.91±0.04^c^ | 0.87±0.05^e^ | 0.51±0.01^b^ | 0.33±0.02^d^ | 3.73±0.05^b^ | 2.67±0.07^e^ | 2.42±0.05^c^ | 1.20±0.05^e^ |
| 96h | 1.58±0.09^d^ | 0.53±0.06^f^ | 0.43±0.05^c^ | 0.31±0.01^d^ | 3.70±0.18^b^ | 1.72±0.13^f^ | 2.01±0.12^d^ | 0.83±0.04^f^ |
| 120h | 0.94±0.04^e^ | 0.28±0.02^g^ | 0.35±0.01^d^ | 0.26±0.05^f^ | 2.65±0.03^c^ | 1.05±0.07^g^ | 1.29±0.04^e^ | 0.54±0.01^g^ |

*Note*. The 2nd leaf from each peanut seedling after 0-h, 12-h, 24-h, 48-h, 72-h, 96-h and 120-h cold treatments was harvested for the determination of chlorophyll content (mg g^-1^ FW). Data are the means ± SD of three independent biological samples. Means denoted by the same letter do not differ significantly according to Tukey's test (p < 0.05). Chl-a = chlorophyll a; Chl-b = chlorophyll b; Chl-a/b = chlorophyll (a/b); Chl-total = chlorophyll (a+b); FW = fresh weight.
