# Supplementary material for: An Advanced Lipid Metabolism System Revealed by Transcriptomic and Lipidomic Analyses Plays a Central Role in Peanut Cold Tolerance
Source: Front Plant Sci. 2020 Jul 21;11:1110. doi: 10.3389/fpls.2020.01110 (PMC7396583; doi:10.3389/fpls.2020.01110)
Supplement: Supplementary file 1 [file DataSheet_1.zip › Supplementary Material/Table S5.docx]

**Table S5** Numbers of different expressed genes (DEGs) in NH5 and FH18.

| **DEG Set** | **All DEGs** | **Up-regulated DEGs** | **Down-regulated DEGs** |
| --- | --- | --- | --- |
| S0 Vs T0 | 225 | 130 | 95 |
| S1 Vs S0 | 8816 | 4287 | 4529 |
| S2 Vs S0 | 6310 | 3620 | 2690 |
| T1 Vs T0 | 8943 | 4639 | 2445 |
| T2 Vs T0 | 6835 | 4390 | 2445 |

*Note*. Total RNA was extracted from three biological replicates. Time points were taken for 0 (T0, S0), 12 (T1, S1), 24 (T2, S2) h after cold stress at 6°C in NH5 (T) and FH18 (S), respectively. The evaluation standard of DEGs: Fold change (FC)≥2 and false discovery rate (FDR)<0.01.
